# Maize green leaf area index dynamics: genetic basis of a new secondary trait for grain yield in optimal and drought conditions

**DOI:** 10.1007/s00122-024-04572-6

**Published:** 2024-03-05

**Authors:** Justin Blancon, Clément Buet, Pierre Dubreuil, Marie-Hélène Tixier, Frédéric Baret, Sébastien Praud

**Affiliations:** 1 UMR GDEC, INRAE, Université Clermont Auvergne, 63000 Clermont-Ferrand, France; 2 Biogemma, Centre de Recherche de Chappes, 63720 Chappes, France; 3 UMR EMMAH, UMT CAPTE, INRAE, 84914 Avignon, France

## Abstract

**Key message:**

Green Leaf Area Index dynamics is a promising secondary trait for grain yield and drought tolerance. Multivariate GWAS is particularly well suited to identify the genetic determinants of the green leaf area index dynamics.

**Abstract:**

Improvement of maize grain yield is impeded by important genotype-environment interactions, especially under drought conditions. The use of secondary traits, that are correlated with yield, more heritable and less prone to genotype-environment interactions, can increase breeding efficiency. Here, we studied the genetic basis of a new secondary trait: the green leaf area index (GLAI) dynamics over the maize life cycle. For this, we used an unmanned aerial vehicle to characterize the GLAI dynamics of a diverse panel in well-watered and water-deficient trials in two years. From the dynamics, we derived 24 traits (slopes, durations, areas under the curve), and showed that six of them were heritable traits representative of the panel diversity. To identify the genetic determinants of GLAI, we compared two genome-wide association approaches: a univariate (single-trait) method and a multivariate (multi-trait) method combining GLAI traits, grain yield, and precocity. The explicit modeling of correlation structure between secondary traits and grain yield in the multivariate mixed model led to 2.5 times more associations detected. A total of 475 quantitative trait loci (QTLs) were detected. The genetic architecture of GLAI traits appears less complex than that of yield with stronger-effect QTLs that are more stable between environments. We also showed that a subset of GLAI QTLs explains nearly one fifth of yield variability across a larger environmental network of 11 water-deficient trials. GLAI dynamics is a promising grain yield secondary trait in optimal and drought conditions, and the detected QTLs could help to increase breeding efficiency through a marker-assisted approach.

**Supplementary Information:**

The online version contains supplementary material available at 10.1007/s00122-024-04572-6.

## Introduction

Maize (*Zea mays* subsp. *mays*) is the most produced crop in the world, and a major staple food in several cultures. However maize yields are stagnating or decreasing in around 30% of its growing areas despite a continuous genetic progress (Duvick 2005; Tollenaar and Lee 2006; Ray et al. 2012; Laidig et al. 2014). Indeed, in some regions, the genetic progress is partially countervailed by climate change (Lobell et al. 2011; Ray et al. 2019). Among climatic limiting factors, drought reduces maize yield of 5–6% globally (Lesk et al. 2016) and climate forecasts indicate that drought risk will increase in several maize growing regions around the world by the end of the century (Harrison et al. 2014; Hoegh-Guldberg et al. 2018; Pörtner et al. 2022) leading to even more serious losses (Webber et al. 2018; Leng and Hall 2019). Drought tolerance has therefore become a crucial goal for maize breeding.

Improving grain yield under drought stress is however a difficult task. Grain yield is a highly complex trait resulting from the interaction of the environment and the plant structural characteristics, physiological processes and regulatory pathways along the whole growth cycle. It thus presents large G × E (genotype × environment) interactions and a low heritability, especially under drought stress, that limit breeding efficiency. Conversely, physiological components of grain yield are simpler traits, which may be more heritable and less prone to G × E interaction. Using such secondary traits in breeding programs, based on a well-identified ideotype, could enhance selection efficiency when drought stress occurs and allow a better understanding of yield establishment in limiting conditions. To this aim, secondary traits must be correlated with yield under drought stress, be more heritable and less prone to G × E interaction than yield, and must be measurable at low cost in small plots in a nondestructive and rapid manner (Bolaños and Edmeades 1996; Bänziger and Lafitte 1997; Araus et al. 2012). Breeding programs incorporating secondary traits in addition to yield, through selection indices, have been shown to be particularly effective in stressful environments (Monneveux et al. 2006), and notably more effective than selection on yield alone (Fischer et al. 1989; Bänziger and Lafitte 1997). Accordingly, genomic selection studies have shown that using secondary traits in trait-assisted approaches could improve predictive ability compared to classical model (Rutkoski et al. 2016; Sun et al. 2017; Sandhu et al. 2021). Ribaut and Ragot (2007) and Beyene et al. (2016) also achieved substantial yield gains under drought conditions by using secondary traits QTLs in marker-assisted selection programs.

The green leaf area index (GLAI), the cumulative area of one leaf face per square meter of soil, plays a major role in light interception, transpiration and CO_2_ exchanges. Its dynamics have thus a predominant impact on grain yield establishment (Monteith 1977; Passioura 1977). It corresponds to the integration, at the canopy level, of the radiation and water use efficiency over the plant cycle, which makes it a promising secondary trait to study drought tolerance. However, its measurement in the field have long been too tedious to use it as a secondary trait. Recent advances now make possible to phenotype hundreds of genotypes in the field for the whole GLAI dynamics (Blancon et al. 2019) and pave the way for its use as a secondary trait to identify and better understand the genetic determinants of drought tolerance.

The benefits of such an approach, both in terms of biological understanding and statistical power, depend on the efforts made to model the temporal evolution of the phenotype (Wu and Lin 2006). The most straightforward approach for detecting longitudinal quantitative trait loci (QTLs) is the discrete approach. It considers the different observations made during the growth cycle as independent traits that are each analyzed through a standard QTL model (Wu and Lin 2006; Hurtado et al. 2012; Würschum et al. 2014; Condorelli et al. 2018). However, the biological interpretation of such analysis is not always obvious because it is difficult to give sense to QTL affecting an arbitrarily chosen point across the curve (Malosetti et al. 2006; Hurtado et al. 2012). Furthermore, measurement dates are often determined by practical rather than biological considerations, and the comparison between different studies is difficult because it is unlikely that measurements were made at identical time points. This method also assumes the independence of the measurements made at different times, which can lead to a loss of power for the QTL detection (Wu and Lin 2006; Malosetti et al. 2006). To account for the observations’ covariance over time, it is possible to integrate them within a multivariate (*i*.*e*. multi-trait) model (Galesloot et al. 2014). However, this method, like the previous one, does not account for the temporal dimension and simply represents the studied phenotype as a set of correlated point measurements. It is also difficult to apply in practice because it requires the adjustment of a large number of parameters, and implies that each genotype is characterized at the same date, which is not always possible (Wu and Lin 2006; Malosetti et al. 2006).

A second family of approaches relies on a two-step analysis (Reymond et al. 2003; Hurtado et al. 2012; Crispim et al. 2015; Campbell et al. 2017). The first step consists in estimating a small number of genotypic parameters that summarize the trait dynamics. These parameters can be computed directly from the raw dynamics (slopes, durations, areas, etc.), or extracted from a temporal model most often using exponential, logistic, or Gombertz functions (van Eeuwijk et al. 2019). These genotypic parameters thus incorporate a temporal dimension and present the advantage to be easily interpretable at the biological level. The second step relies on the use of these parameters as response variables in a classical GWAS model. Nevertheless, because these genotypic parameters are often correlated, a joint analysis in a multivariate QTL detection model could better distinguish the effects of markers on each trait, and increase testing power (Campbell et al. 2017). Although this method has been shown to be effective, the quality of the results obtained remains dependent on the accuracy of the estimation of genotypic parameters (Verbeke and Molenberghs 2009). A loss of information between the two steps is also inevitable and may result in a loss of statistical power (Campbell et al. 2019).

Methods that combine temporal modeling and QTL detection in one step, grouped under the functional mapping terminology, have also been proposed, such as the use of mixture models or nonlinear mixed models (Ma et al. 2002; Wu and Lin 2006; Malosetti et al. 2006; Li et al. 2010). However, the use of parametric models may be too restrictive to model the temporal evolution of some traits, including leaf area and senescence (van Eeuwijk et al. 2019). Random regression, using covariance functions (spline or polynomial functions), then offers a nonparametric alternative to model the dynamics of the trait over time, while considering the temporal covariance between each measurement (Kirkpatrick et al. 1990; Wu and Lin 2006; Ning et al. 2017; Campbell et al. 2019). This approach has been widely used in animal genetics (Huisman et al. 2002; Kranis et al. 2007; Howard et al. 2015; Ning et al. 2017, 2018) but is still rare in plant genetics. In plants, it is mainly applied in genomic selection (Sun et al. 2017; Campbell et al. 2018), with the exception of Campbell et al. (2019) that published the first GWAS using a random regression model performed on a major crop (rice). This approach is however highly computationally demanding (Ning et al. 2017; Moreira et al. 2020).

The main objective of the present study was to explore the genetic determinism of maize GLAI dynamics in drought and optimal conditions. We also aimed to better understand its link with grain yield, and to evaluate the potential of GLAI dynamics as a secondary trait in selection. For this, we evaluated a multi-parent advanced generation inter-cross (MAGIC) panel in four environments with contrasted hydric regimes. We also phenotyped the GLAI dynamics over the whole maize life cycle using a multispectral camera mounted on a UAV (unmanned aerial vehicle). A simple physiological GLAI model was then used to derive the parameters of the dynamics. To appraise the potential of GLAI dynamics as a secondary trait in selection, its sensitivity to G × E interaction was compared to that of yield and its components. We then applied a univariate and a multivariate two-step GWAS approach based on these parameters to identify and characterize the GLAI dynamics genetic determinants.

## Materials and methods

### Plant material, genotyping and relatedness

We used a broad diversity panel of doubled haploid (DH) maize lines derived from a MAGIC population. This population originated from a funnel cross with 16 parents chosen among historical lines that are representative of the genetic diversity of temperate material (Buet et al. 2013). To limit confounding effects due to precocity differences, certain DH lines were discarded to narrow the range of flowering times, with 95% of the panel flowering within an eight-day period when evaluated in a hybrid context.

The 16 parents of the panel were sequenced with the Illumina HiSeq 2000 at a 12–16 *X* depth of coverage. The 324 DH lines were genotyped with the Illumina 50 K array (Ganal et al. 2011) and the 600 K Affymetrix array (Unterseer et al. 2014). Using the genotyping data, parental haplotypes were reconstructed with a modified version of the *R* package *qtl* (Broman et al. 2003; R Core Team 2017), then the parental origin was inferred for each allele in every DH line. Based on parental mosaic and sequencing data for each of the 16 parents, the panel was genotyped in silico, resulting in around 17 million single nucleotide polymorphisms (SNPs), that were narrowed down to 7,806,995 SNPs after filtering out any with missing data in parents or a low minor allele frequency (3.5% threshold).

The genomic relatedness between individuals was estimated from the Illumina 50 K array genotyping data. Removal of SNPs with missing data or at biased SYNGENTA loci (Ganal et al. 2011) resulted in 32,466 SNPs evenly distributed on the 10 chromosomes, which were then used to compute a global identity-by-state (IBS) kinship matrix and 10 leave-one-chromosome-out (LOCO) IBS kinship matrices (Rincent et al. 2014) in the *R* package *GAPIT* (Lipka et al. 2012).

### Agronomic evaluation of maize hybrids

DH lines were crossed with the tester line MBS847 and 360 of the resultant hybrids were evaluated in the field in 2016 and 347 were evaluated in 2017, 330 being common to both years. Each year, a well-watered (WW, irrigated) trial and a water-deficient (WD, rain-fed with monitored irrigation) trial were carried out in Saint-Paul-lès-Romans, close to Romans-sur-Isère, France (45°04′06″N, 5°08′01″E). The trials were laid out as alpha-lattice designs with two replicate blocks in each treatment. The microplots were sown at a density of 9 plants/m^2^ with two rows per microplot, a 0.8 m row spacing and a row length of 6 m. The combination of the two years and two water treatments were considered as four environments 16STPAULWD, 16STPAULWW, 17STPAULWD, and 17STPAULWW. More details about climatic conditions and agronomic practices are given in Blancon et al. (2019).

In the 16STPAULWD environment, as evidenced by water balance analysis (Fig. S1), drought stress occurred for a short period during flowering and more severely during grain filling leading to 38% grain yield loss compared to 16STPAULWW. In the 17STPAULWD environment, stress only occurred during flowering (Fig. S1) leading to 20% grain yield loss compared to 17STPAULWW. In each trial, we measured grain yield (GY, in q.ha^−1^) and thousand kernel weight (TKW, in g), both adjusted to 15% humidity. We computed kernel number per square meter (KN, in grain.m^−2^), and scored the female flowering date (FF, in °C.j) and the anthesis to silking interval (ASI, in °C.j).

Between 2014 and 2017, the performance of the maize hybrid panel was evaluated in 11 field trials under WD conditions in five locations: Blois, Saint-Paul-lès-Romans and Nérac in France, Szeged in Hungary and Graneros in Chile. More details on trial conditions can be found in Table S1. These 11 environments include 16STPAULWD and 17STPAULWD, described above. GY data was obtained in each of these 11 experiments (GY_11_).

### Characterization of GLAI dynamics and derived traits

#### UAV model-assisted phenotyping of GLAI dynamics

We flew a hexacopter UAV mounted with a six-band multispectral camera nine times over the 2016 trial plots and eleven times over the 2017 trial plots. The multispectral camera is composed of six cameras with 10-nm spectral resolution bands centered, respectively, at 450, 532, 568, 675, 730, and 850 nm. Images were recorded continuously during the flight at a frequency of 1 Hz, and the integration time was adjusted automatically to minimize saturation and maximize the range of variation. The reflectances recorded in the first five bands were normalized by dividing the values by the reflectance measured in the near-infrared to limit the effect of illumination variations during the flight. More details about the processing of multispectral images are given in Fig. S2 and in Blancon et al. (2019).

For each flight, we calibrated empirical relationships linking ground level measurements of GLAI achieved over a small sample of microplots to normalized reflectances. We then estimated GLAI value at each time point from these transfer functions for each microplot of the trials.

Finally, we used a simple, physiologically-based dynamics model to fit the GLAI estimates and predict the continuous GLAI dynamics for each microplot over the whole season in steps of one growing degree day with a 6 °C base (GDD6). More details can be found in Blancon et al. (2019).

#### Estimation of traits describing GLAI dynamics

To describe the GLAI dynamics, we derived integrative traits over five physiological distinct stages: an early vegetative (EV), late vegetative (LV), flowering (*F*), slow senescence (SS) and rapid senescence phase (RS).

**Fig. 1:**
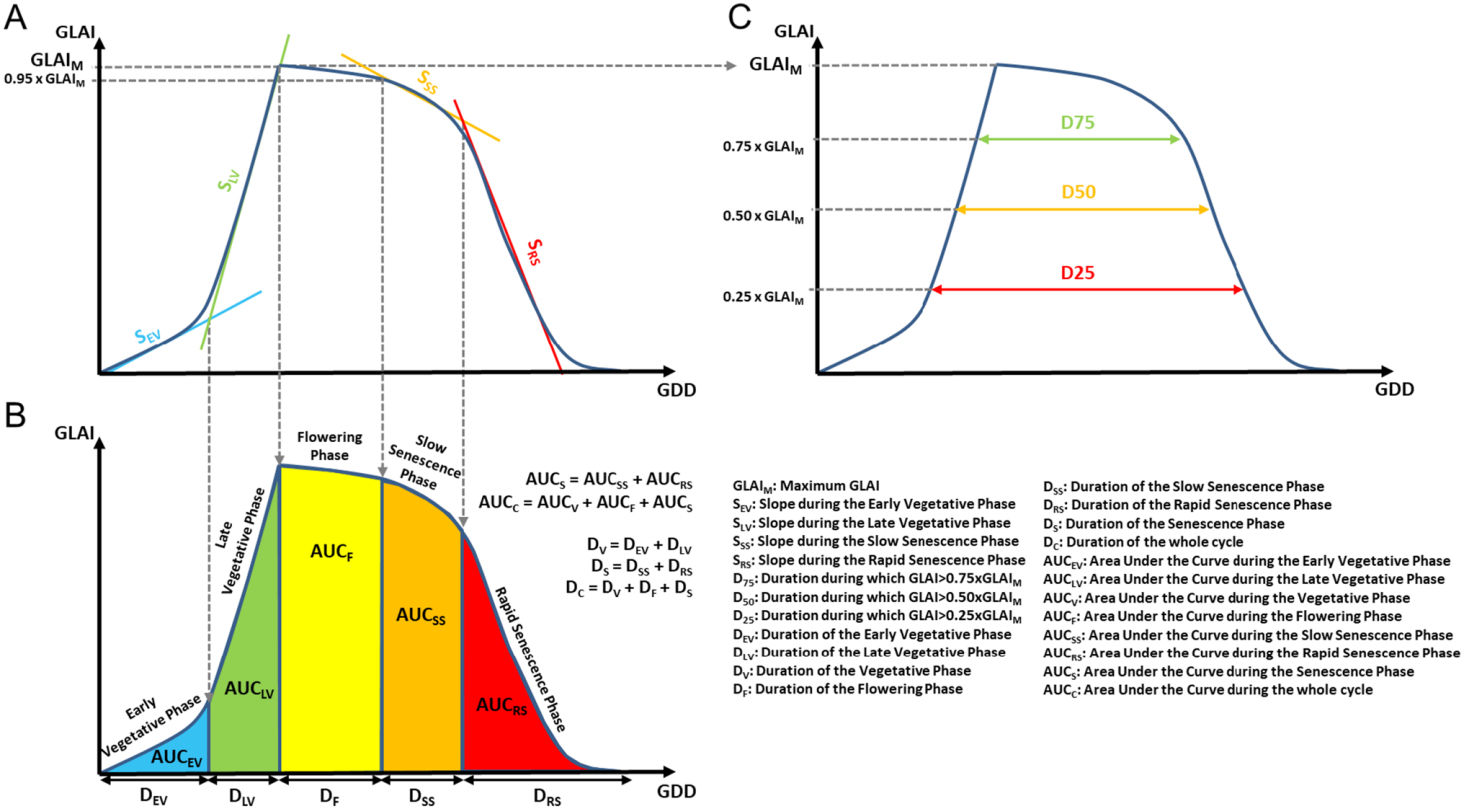
Estimation of 24 parameters derived from maize GLAI dynamics. **A** Two bent-cable regressions were fitted, one during the vegetative phase and the other during the senescence phase, to estimate four slopes (S_EV_, S_LV_, S_SS_, S_RS_) and to define five distinct phases during the development cycle. **B** The phases previously defined were used to derive eight areas under the curve (AUC_EV_, AUC_LV_, AUC_V_, AUC_F_, AUC_SS_, AUC_RS_, AUC_S_, AUC_C_) and the eight corresponding durations (D_EV_, D_LV_, D_V_, D_F_, D_SS_, D_RS_, D_S_, D_C_). Finally, the maximum GLAI reached during the cycle (GLAI_M_) was extracted from the model and then used to derive three additional durations (**C**: D_75_, D_50_, D_25_) (color figure online)

We delimited the first two phases with a bent-cable regression (*R* package *SiZer,* Sonderegger (2022)) fitted to the GLAI dynamics between shoot emergence (when GLAI becomes positive) and the time when GLAI achieves its maximum (Fig. 1, *S*_EV_ and *S*_LV_). The abscissa of the intersection between the two linear parts of the regression defines the limit between the EV and LV phases. The flowering phase extends from the time when GLAI reaches its maximum until the onset of senescence, here defined as the time when the GLAI becomes less than 95% of the maximum GLAI. We delimited the last two phases by fitting a bent-cable regression to the GLAI dynamics between the start of senescence and complete senescence (when the GLAI becomes zero) (Fig. 1, *S*_SS_ and *S*_RS_). The abscissa of the intersection between the two linear parts of this regression delimits the SS from the RS phase. A set of 24 traits counting slopes, durations, and areas under the curve were finally computed from these five phases, alone or combined (Fig. 1). These traits were extracted for each microplot of the four trials.

### Adjusted means, heritability, and variance decomposition

For each combination of trait, year and treatment, we used a linear mixed model with block effect, genotype effect and autocorrelated errors (Model S1) to estimate the adjusted means. The same model, with random genotype effect, was used to compute generalized heritability (Eq. S1, Cullis et al. 2006).

To analyze variance components at the network scale, we used a multi-environmental model with random genotype and G × E interaction effects (Model S2). Each random effect was tested with a restricted likelihood-ratio test comparing the full model and the null model without the considered effect. The variance components were extracted to be compared, and the standard deviation was expressed as a percentage of the mean $\mu$ (CV_*g*_). All linear models were fitted using the *R* package *asreml-R v3.2* (Butler et al. 2009).

### Trait correlation analysis

To get an overall picture of correlations between traits at the network scale, we performed a principal component analysis (PCA) with the *R* package *FactoMiner* (Lê et al. 2008), based on standardized adjusted means of GLAI and agronomic traits from the four environments. From the PCA results, six GLAI traits were retained for further analysis because they were representative of the panel diversity and characterize complementary biological processes: D_V_, D_S_, GLAI_M_, AUC_V_, AUC_F_ and AUC_S_, the duration of the vegetative phase, the duration of the senescence phase, the maximum of GLAI, the area under the curve during the vegetative, the flowering and the senescence phase respectively.

To better understand the link between GLAI dynamics and GY we evaluated the phenotypic correlation (*r*_*p*_) by computing the Pearson correlation coefficient between these six GLAI traits and yield components, and tested it with a Student test in each of the four environments.

### Genome wide association studies

To identify the genetic determinants of GLAI dynamics, we used two GWAS methods: a univariate approach (*M*_UV_) and a multivariate approach (*M*_MV_). For both approaches, the LOCO method was used to avoid proximal contamination (Rincent et al. 2014).

#### Univariate approach

A first association study was done trait by trait for each environment of the network, using an univariate model. The six chosen GLAI traits and the five agronomic traits were analyzed with this univariate approach. Model S3 was fitted with *FaST-LMM v2.07* (Lippert et al. 2011), and the null hypothesis $H_{0} : \beta = 0$ was tested with a likelihood-ratio test.

#### Multivariate approach

In the second association study, we analyzed several GLAI and agronomic traits jointly in the same multivariate model for each environment. Model S4 was fitted using *GEMMA* software (Zhou and Stephens 2014) for $t$ = 8 traits namely AUC_V_, AUC_F_, AUC_S_, GLAI_M_, D_V_, D_S_, GY and FF. Because of convergence issues, it was not possible to include other agronomic traits in the model. The null hypothesis $H_{0} : \beta_{1} = \cdots = \beta_{d} = 0$ was tested with a Wald test. *GEMMA* was also used to estimate genetic correlations (*r*_g_) between these eight traits.

#### SNP clustering

To correct for multiple testing and account for the false discovery rate (Storey 2002), we computed a *qvalue* for each test with the *R* package *qvalue* (Storey et al. 2021). SNPs with $q\;{\text{value}} \le 0.05$ were considered as significantly associated.

For the multivariate approach, rejecting the null hypothesis ($H_{0} : \beta_{1} = \cdots = \beta_{d} = 0$) does not give information about the trait(s) associated with the significant SNP. Significant SNPs effect for each trait were estimated with their standard deviation. The significance of these effects was tested a posteriori with a Wald test ($\alpha = 0.01$) for each trait, to assign each significant SNP to its trait(s).

For an environment-chromosome-trait combination, associated SNP were clustered with the method proposed by Cormier et al. (2014) with a linkage disequilibrium threshold of $R^{2} \ge 0.6$. For a given combination, overlapping clusters were aggregated. Three different types of clusters were computed: *M*_UV_ clusters built from SNP detected with *M*_UV_, *M*_MV_ clusters built from SNP detected with *M*_MV_; and *M*_ALL_ clusters built from SNP detected with *M*_UV_ or *M*_MV_. Clusters longer than 30% of the chromosome length were discarded as artifacts. Each cluster was finally described by its most strongly associated SNP (peak SNP with the smallest *pvalue*) and the interval covered by its SNP components.

#### QTL identification

We used a multi-environment multilocus backward selection model to identify SNP clusters playing a major role in the genetic determinism of each trait in the trial network (Model S5). Initially, the effects of all candidate clusters were incorporated in Model S5. The effect of each cluster was tested with a Wald test when it was added last to the model. The cluster with the least significant effect was iteratively removed, until all remaining clusters had been declared significant ($\alpha = 0.01$). After the backward elimination, the remaining clusters were called QTL. QTL effects were estimated in each environment with Model S5, and tested a posteriori with a Wald test ($\alpha = 0.01$) to identify the environment(s) in which each QTL significantly affects the considered traits. Finally, we defined the QTL limit as the initial cluster interval extended by the local linkage disequilibrium extent at each extremity. QTL effects were defined as B73 allele effect, a parental line of the panel.

#### QTL colocalization

To decipher the genetic link between the traits, we studied QTL colocalization for every combination of trait and environment. Colocalization was defined as, at least, two overlapping QTL.

### Grain yield prediction from GLAI QTL

A multi-environment model was used to confirm the utility of GLAI as a yield secondary trait under drought stress conditions using GY_11_ data from the eleven-environment WD trial (“Agronomic evaluation of maize hybrids” section). We quantified the proportion of GY_11_ variance explained by the GLAI QTLs with a backward selection approach. This led to a list of GLAI QTLs that significantly impact GY at the GY_11_ network scale. As this approach was applied to the GLAI QTLs resulting from an initial backward elimination (“QTL identification” section), and not to all the clusters detected, a less conservative threshold was applied (*α* = 0.05). A Wald test ($\alpha = 0.05$) was used a posteriori to identify which QTL were significant in each environment.

## Results

### Six parameters of GLAI dynamics are good descriptors of maize diversity and environmental constraints

**Fig. 2:**
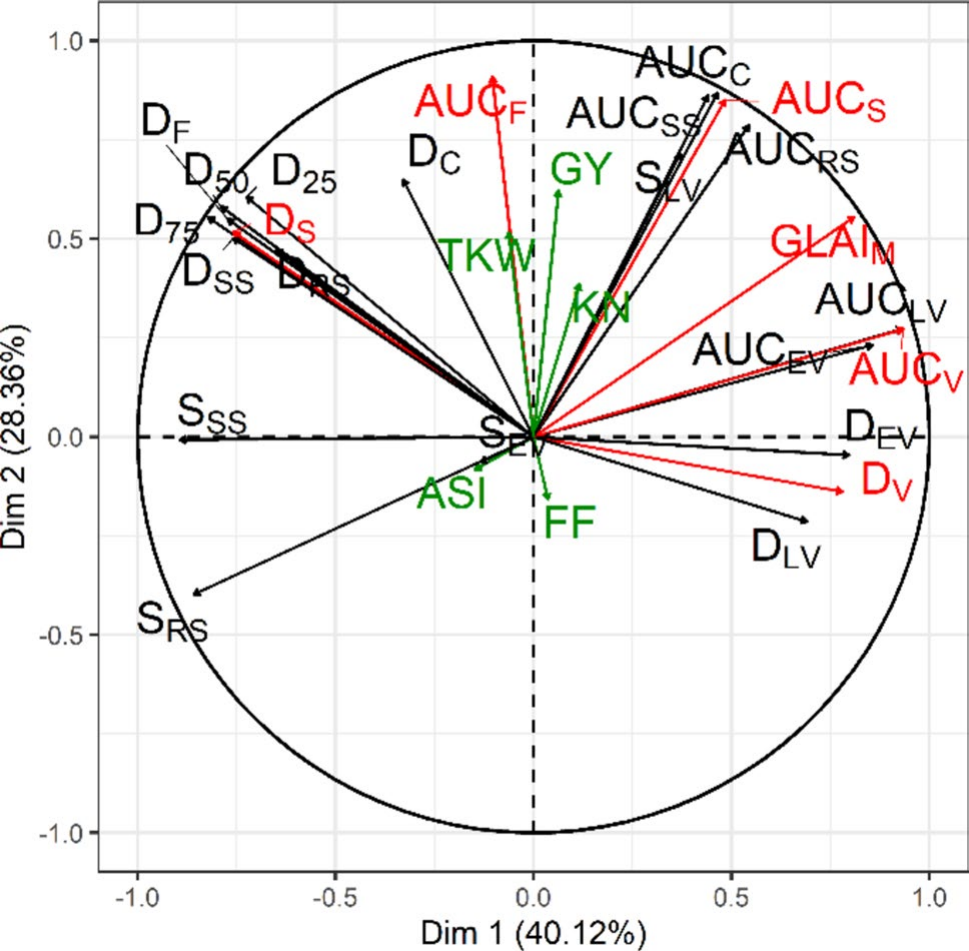
Correlation circle from principal component analysis of 29 maize traits. A normalized PCA was done on the adjusted means for five agronomic traits (green arrows) and 24 GLAI traits (black and red arrows) in 324 maize hybrids in four environments. The first two axes (Dim) are plotted together with their proportion of explained variance. For trait abbreviations see the “Material and methods” section and Fig. 1. Six GLAI traits were chosen as representing the panel diversity (red arrows) (color figure online)

To get a global picture of GLAI and agronomic traits correlation in the trial network, we ran a principal component analysis of the 24 GLAI related and the 5 agronomic traits in the four environments together (Fig. 2). The first two PCA axes explained respectively 40% and 28% of the panel diversity. The first axis differentiated genotypes with a long vegetative phase (D_V_) from those with a long senescence phase (D_S_, S_SS_). The second axis was more related to the GLAI maximum (GLAI_M_) and the flowering phase (AUC_F_, AUC_C_). In this plane, the GLAI traits are better represented than the agronomic traits. Six traits—D_V_, D_S_, GLAI_M_, AUC_V_, AUC_F_ and AUC_S_—are particularly well correlated with the axes, each showing a Pearson *r*^2^ > 0.57 with one of the two axes. These traits also captured most of the information carried by the other GLAI traits at the network scale, for example, D_S_ for D_SS_, D_RS_, D_*F*_, D_25_, D_50_, and D_75_, or AUC_S_ for AUC_*C*_, AUC_SS_, AUC_RS_ and S_LV_ (Fig. 2). The aforementioned six traits were therefore chosen as good descriptors of the GLAI diversity in the panel. Of the agronomic traits, GY is the best represented, mainly by the second component (*r*^2^ = 0.39). GY is thus more correlated to the GLAI_M_ and the AUC traits than to the duration of the vegetative or senescence phase. Conversely, grain yield components TKW and KN were more weakly linked with the GLAI traits, while flowering traits FF and ASI, are poorly related to GLAI dynamics and GY on the whole network.

**Fig. 3:**
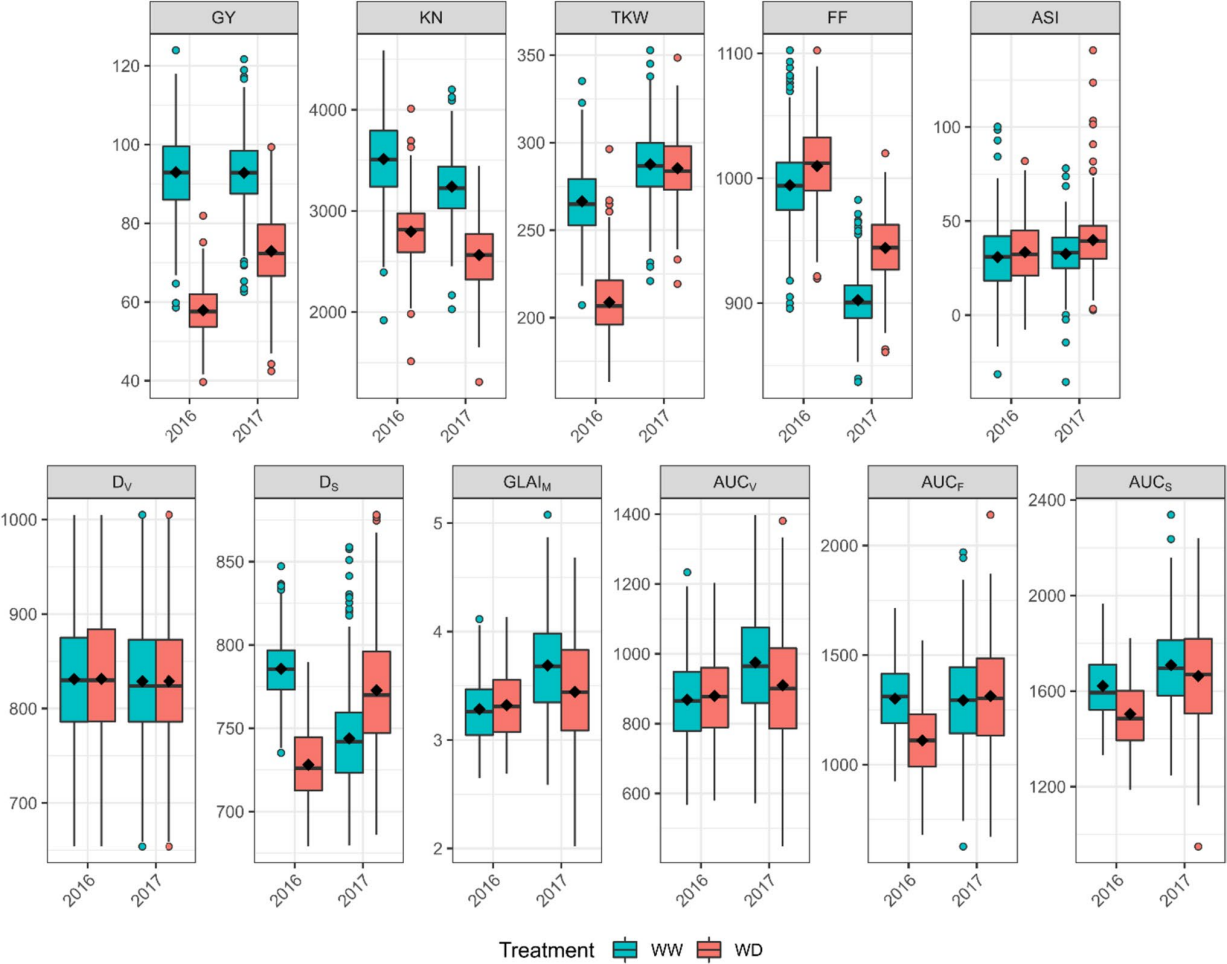
Drought stress impact on five agronomic traits and six modeled GLAI traits representative of maize diversity. The agronomic traits are GY (q.ha^−1^), KN (kernels.m^−2^), TKW (g), FF (GDD6) and ASI (GDD6). The GLAI traits are D_V_ (GDD6), D_S_ (GDD6), GLAI_M_ (m^2^.m^−2^), AUC_V_ (m^2^.m^−2^.GDD6), AUC_F_ (m^2^.m^−2^.GDD6), and AUC_S_ (m^2^.m^−2^.GDD6). Boxplots were constructed from the adjusted means of traits measured in 324 maize hybrids grown in the field in well-watered (WW) and water-deficient (WD) conditions. Boxes represent the inter-quartile range and whiskers extend to 1.5 times the inter-quartile range. Medians (horizontal lines inside the box), means (diamond symbols), and outliers (circle symbols) are indicated for each trait in each environment (color figure online)

Environmental conditions affected differently GLAI and agronomics traits in 2016 and 2017 (Fig. 3). Drought stress affected agronomic traits in the same direction in 2016 and 2017, with decreases in GY, KN and TKW, and a delay in FF, leading to a slight increase in ASI. TKW was barely impacted in 2017 by the drought stress as it occurred during the flowering phase. GLAI traits were more affected by the different timing of the drought stress in 2016 and 2017, which reveal contrasting physiological constraints between the two years. GLAI traits related to the vegetative phase were less affected by drought stress than those related to the magnitude or senescence phase, perhaps because drought had no impact on the duration of the vegetative phase (D_V_ Fig. 3, D_EV_ and D_LV_ Fig. S4). Indeed, drought stress began a few days before flowering in 2016 and 2017, so had only a marginal impact on vegetative processes. However, drought stress was early enough to decrease the dynamics amplitude (GLAI_M_) in 2017, mainly by reducing leaf area (Blancon et al. 2019), and thus AUC_V_ and AUC_S_. The lower GLAI_M_ in 17STPAULWD seems to have led to a delayed senescence (D_S_) that could be explained by better light distribution in the canopy strata (Borrás et al. 2003; Huang et al. 2017; Yang et al. 2019). The drought effect on GLAI_M_ and the consequences on D_*S*_ compensated each other, so the decrease in AUC_S_ in 2017 was only slight. Conversely, in 2016 the drought stress mainly occurred during grain filling, which curtailed the stay-green phase (D_*F*_ and D_S_, Fig. 3, Fig. 1), so AUC_F_ and AUC_S_ were smaller.

The relationships between the 11 traits are likely to vary under different conditions, so Pearson correlation coefficients (*r*) were calculated between pairs of traits analyzed within each environment (Fig. S3). The most strongly correlated traits were D_V_ and D_S_ (*r* values range from 0.23 to − 0.83), D_V_ and AUC_V_ (*r* from 0.77 to 0.92), D_*S*_ and AUC_V_ (*r* from − 0.22 to − 0.73), GLAI_M_ and AUC_V_ (*r* from 0.84 to 0.90), GLAI_M_ and AUC_S_ (*r* from 0.89 to 0.95), and GY and KN (*r* from 0.64 to 0.86). There was therefore a strong compensation between the duration of the vegetative phase and the duration of the senescence phase, as described by the first axis of the PCA. Moreover, the AUC traits seemed more dependent on the amplitude of the dynamics (GLAI_M_) than on the duration of the phases. Correlations between yield and GLAI traits were strongest for AUC_S_, AUC_F_ and GLAI_M_ (*r* from 0.23 to 0.50). Globally the latter three traits were more closely correlated to yield than were the flowering traits FF and ASI. TKW was never significantly correlated with GLAI traits, except in the 16STPAULWD environment where it was correlated with AUC_F_ (*r* = 0.16) and with the duration of senescence (*r* = 0.26). The correlation between TKW and GY was also the strongest (*r* = 0.33) in 16STPAULWD where the drought stress that occurred during grain filling had an impact on the duration of senescence.

### *GLAI traits exhibit limited G* × *E interaction*

The 11 traits analyzed all showed significant genetic effects (Table 1). The genetic variance in GY is noteworthy with CV_g_ = 7%. All GLAI traits, except D_S_, showed more genetic variance (higher CV_g_) than GY, while ASI was the only agronomic traits showing more variance than GY. The two traits with the least genetic variance were D_S_ and FF with CV_g_ values around 2.5%. On the contrary, ASI and AUC_V_ had the strongest genetic variances, with a CV_g_ of 23% and 15% respectively. All agronomic traits had a high generalized heritability (*H*^2^ > 0.8) except ASI, but GLAI traits were even more heritable. The G × E interaction was significant for all traits, except D_V_. Apart from D_S_, G × E interaction is smaller in GLAI traits (1–11% of the genetic variance $\sigma_{g}^{2}$) than in agronomic traits (12–54% of the genetic variance). The G × E interactions of D_S_ and GY were however similar (52% of the genetic variance).

**Table 1:** Variance decomposition of six modeled GLAI traits and five agronomic traits in the maize trial network

Trait	Unit	Min–Max	Mean	${\varvec{\sigma}}_{{\varvec{g}}}^{2}$		CV_g_	${\varvec{\sigma}}_{ge}^{2}$		${\varvec{\sigma}}_{{{\text{ge}}}}^{2} /{\varvec{\sigma}}_{{\mathbf{g}}}^{2}$ (%)	*H*^2^
D_V_	GDD6	654–1005	829	4215	***	7.83	3.91 10^–8^	n.s	0.00	0.91
D_S_	GDD6	620–892	790	475	***	2.76	246	***	51.86	0.85
GLAI_M_	m^2^.m^−2^	1.44–5.66	3.20	0.10	***	9.64	2.4110^–3^	***	2.53	0.88
AUC_V_	m^2^.m^−2^.GDD6	362–1502	844	15,296	***	14.65	188	***	1.23	0.89
AUC_F_	m^2^.m^−2^.GDD6	512–2220	1287	23,764	***	11.98	2545	***	10.71	0.88
AUC_S_	m^2^.m^−2^.GDD6	681–2632	1589	17,779	***	8.39	917	***	5.16	0.87
GY	q.ha^−1^	21–134	91	43.6	***	7.25	22.5	***	51.67	0.83
KN	kernels	954–4964	3446	69,258	***	7.64	21,847	***	31.54	0.82
TKW	g	140–367	265	240	***	5.85	56.2	***	23.40	0.85
FF	GDD6	823–1140	1007	514	***	2.25	63.3	***	12.32	0.85
ASI	GDD6	− 87–187	31	51.2	***	22.77	27.6	**	53.86	0.50

Min, Max and Mean were computed from the adjusted means of the traits of 324 hybrids over four environments. $\sigma_{{\text{g}}}^{2}$, genetic variance; CV_g_, genetic coefficient of variation; $\sigma_{ge}^{2}$, G × E interaction variance; $\sigma_{{{\text{ge}}}}^{2} /\sigma_{{\text{g}}}^{2}$, ratio of G × E interaction variance to genetic variance (%); *H*^2^, generalized heritability

***pvalue ≤ 0.001; **pvalue ≤ 0.01; *pvalue ≤ 0.05; n.s., *p*value > 0.05.

### Different associations between polymorphisms and traits in environments found with univariate and multivariate approaches

**Fig. 4:**
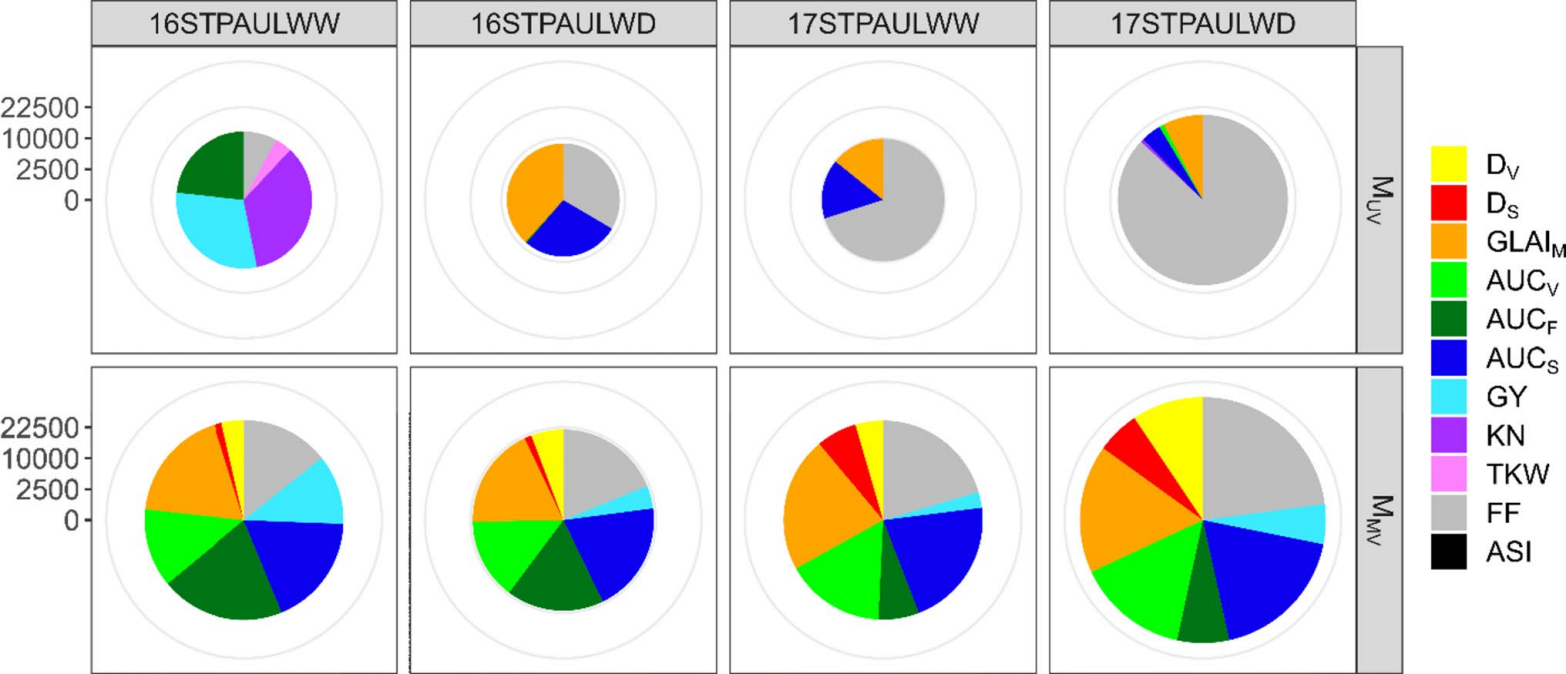
Number of associations detected in univariate (M_UV_*)* and multivariate (M_MV_*)* GWAS for 11 traits measured on 324 maize hybrids in four environments. The diameter of each circle is proportional to the total number of associations detected for each approach in that environment. Associations with KN, TKW and ASI were not tested in the M_MV_ approach (color figure online)

In a GWAS, a total of 7,806,995 SNPs were tested at 5% false discovery rate threshold for each environment-trait combination with two methods. With the univariate approach (M_UV_) 49,459 significant associations (combinations between a trait, a SNP and an environment) were detected for all 11 analyzed traits. For all environments combined, FF represented 55% of the associations detected, GLAI_M_ 12%, and AUC_F_, AUC_S_, GY and KN between 5 and 10% each (Fig. 4). No association was found for D_V_, D_S_ or ASI. Most other traits had at least one association in two different environments, but not GY, TKW and AUC_V_. Flowering was the only trait with associations in all four environments.

With the multivariate approach (M_MV_) 33,043 associated SNPs were revealed, but 9% of them could not be assigned to a trait with the a posteriori Wald test (*α* = 0.01). Thus, 113,164 significant associations were detected for eight traits in M_MV_, which compares to 44,572 associations for the same eight traits for M_UV_. All eight traits exhibited significant associations in each environment in M_MV_, which contrasts sharply with the univariate approach (Fig. 4). The distribution of associations between each trait and environment was also much more even. For instance, FF, GLAI_M_, AUC_V_ and AUC_S_ each represented 15–20% of associations.

It must be noted that in the univariate approach, most of the associated SNPs (86%) were associated with only one trait in a given environment, 11% with two, 3% with three and 0.4% with four environments. In contrast, the multivariate approach detected many more SNPs associated with several traits in a given environment. Specifically, 79% of SNPs were associated with 2–8 traits, and 23%, 22% and 17% of SNPs were associated with 4, 5 and 6 traits, respectively.

Most associations were specific to the GWAS model used, with 54% specific associations in M_UV_ and 85% in M_MV_. Only 13% of GLAI trait associations were detected by both models (Fig. S5), 9% of GY associations and 19% FF associations.

### GLAI traits have a simpler genetic architecture than yield

Significant SNPs were clustered for each trait-environment combination based on linkage disequilibrium. Within mixed clusters (i.e., defined as a mixture of SNPs detected by M_UV_ and M_MV_), most associated SNPs in each metacluster were from the multivariate approach (81% of cases).

Among the 1444 clusters identified, 19 were longer than 10% of the corresponding chromosome. The lengths of the clusters were quite short compared to the large number of associated SNPs, with 75% of clusters shorter than 0.25 Mbp (90% < 3.5 Mbp).

As expected, for a given trait, the number of clusters identified depends strongly on the number of associated SNPs (Table S2). However, the 25,591 SNPs associated with FF in 17STPAULWD were grouped into just 66 clusters. On the contrary, the 2,079 SNPs associated with GY in the same environment were grouped into as many as 96 clusters, the maximum detected in this study for a single trait in a given environment. This is consistent with the highly polygenic architecture of yield.

A backward elimination approach was applied to the large number of genomic regions detected. This had the twofold advantage of limiting the risk of false positives and reducing the number of regions to analyze. Thus, only a third of the clusters were kept with 1 to 20 QTLs depending on the trait and environment considered, thus accounting for 475 QTL in total (Tables S2 and S3). Moreover, the multi-environment backward selection model can also be used to identify QTLs in environments where they had not been initially detected (Table S2). While most QTLs are concentrated on chromosomes 1, 3, 4, 5 and 8, there are at least 15 QTLs on each chromosome.

In each environment, the average proportion of phenotypic variance explained by a QTL is 4%, with a maximum of 11%, and no major differences between GLAI traits and agronomic traits. Nevertheless, among the 16 QTLs explaining less than 1% of the phenotypic variance, eight were yield QTLs. Some differences are also apparent at the network scale: the mean and maximum proportion of variance explained by QTL were, respectively, 3.5% and 9.5% for GLAI traits, 2.3% and 5.2% for yield and its components, and 2.8% and 6.1% for flowering. This confirms that GLAI traits have a weaker G × E interaction than yield and flowering.

**Fig. 5:**
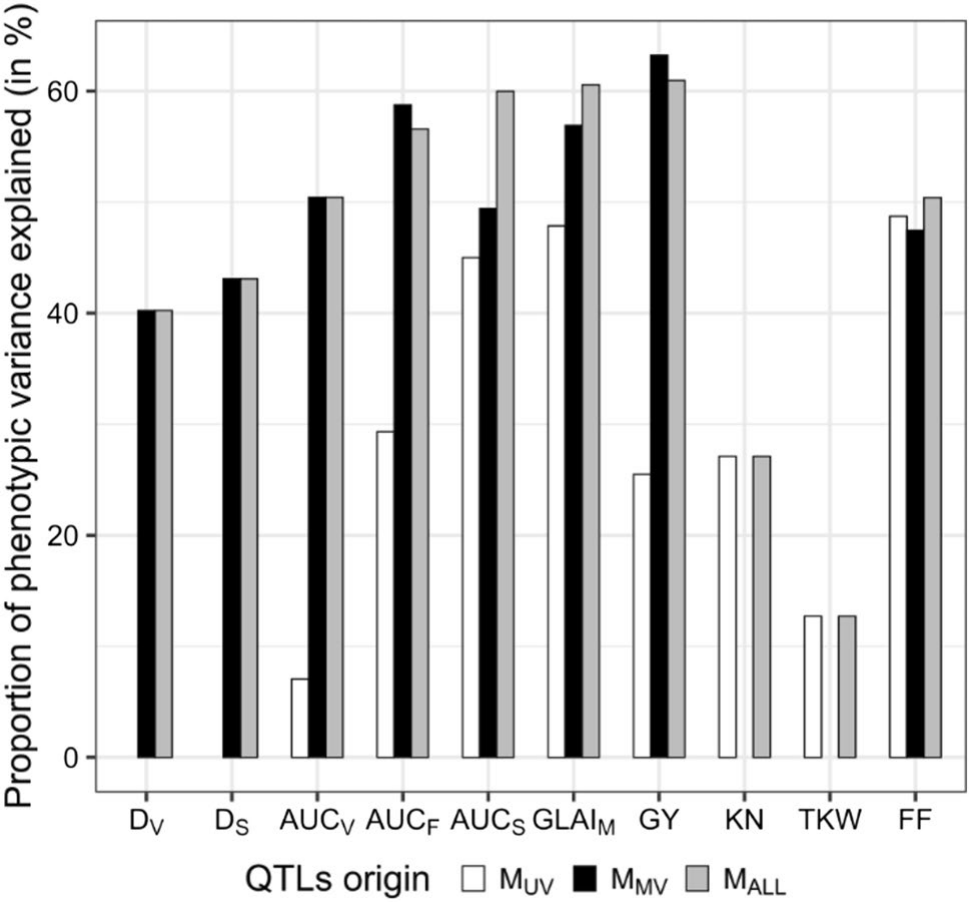
Proportion of phenotypic variance explained by all the QTLs detected on 324 maize hybrids evaluated in four environments. For each trait, we compared the variance explained by the QTLs from a univariate GWAS approach (M_UV_), the QTLs from a multivariate approach (M_MV_), and the QTLs from M_ALL_ built from the combination of SNPs detected by M_UV_ and M_MV_. Associations with KN, TKW were not tested with M_MV_. No association was found for ASI

Overall, the QTLs detected captured 13–61% of the phenotypic variance depending on the trait considered (Fig. 5, M_ALL_). To evaluate the respective contributions of M_UV_ and M_MV_ approaches to explain the phenotypic variance, two new sets of QTLs were selected by backward elimination, from the SNPs detected independently by each approach. It appeared that the QTLs detected by M_UV_ explained less of the variance in all the traits analyzed, except FF. Indeed, with the M_MV_ approach *r*^2^ values for the six GLAI traits and GY were 4–43% higher. The combined use of M_UV_ and M_MV_ brought little gain compared to M_MV_ alone, at most + 11% for AUC_S_.

### GLAI QTLs show many colocalizations

According to the genomic sequence, many QTLs are colocalizing suggesting significant pleiotropy or genetic linkage between the analyzed traits, in particular the GLAI traits. Indeed, 53 to 89% of GLAI QTLs colocalized with QTLs for at least one other trait in one or more environments, compared to only 27–43% of agronomic QTLs. Moreover, QTLs of the same trait in different environments often colocalized, implying that trait- and environment-specific QTLs are rare (Fig. S6). It is also noteworthy that most of the QTLs had similar effects in the four environments and it was rare that the direction of the effect reversed (Figs. S4 and S7).

Overall, GLAI traits shared a significant proportion of QTLs with same-direction effects, especially AUC_V_, AUC_F_ and AUC_S_, but also AUC_V_ and D_V_ (Fig. S6). The colocalizations between GLAI traits and GY or FF, distributed over one to seven genomic regions, were rarer. Despite this marked trend, there were some QTL colocalizations with opposite effects for almost all trait pairs.

Yield QTLs detected under drought stress showed fewer colocalizations than those detected under optimal conditions, especially for 16STPAULWD where the stress lasted longer (Fig. S6). However, interestingly, in both stressed environments, some yield QTLs colocalized with GLAI QTLs detected under optimal conditions. Additionally, yield and flowering QTLs rarely colocalized, with only three common regions, each affecting both traits in the same direction. In particular, there was a single colocalization between yield (17STPAULWD) and flowering (16STPAULWD) under stressed conditions.

Some traits showed only colocalizations with opposite effects, such as D_V_ and D_S_, AUC_V_ and D_S_, and GLAI_M_ and D_S_ (Fig. S6), which is consistent with the mainly negative phenotypic (Fig. S3) and genetic (Fig. S8) correlations between these pairs of traits. Notably, three colocalizations with opposite effects were detected for AUC_S_ and GY versus only one with effects in the same direction. This trend is surprising because it contradicts the marked positive correlations between AUC_S_ and GY at the phenotypic and genetic level and probably reflects the weaknesses of the QTL colocalization method, or the backward elimination approach for the selection of the final QTL list.

### GLAI QTLs can explain yield variability under drought stress conditions

**Fig. 6:**
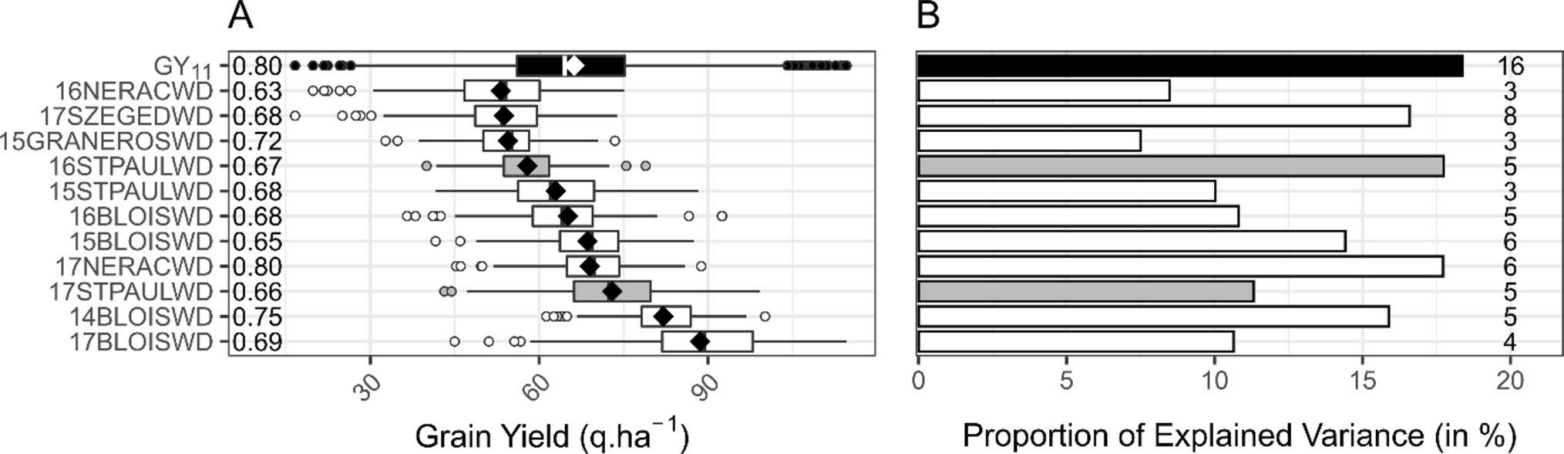
Grain yield variability explained by GLAI QTLs in the GY_11_ drought stressed network. **A** Effect of a wide range of drought stress on yield of 324 hybrids measured in eleven environments. The black boxplot corresponds to the whole GY_11_ network, which is composed of the eleven stressed environments detailed below. The gray boxplots correspond to the two WD trials used to identify the GLAI QTLs then used in the backward selection approach to explain grain yield variability in the whole network. Boxplots were constructed from the adjusted means. The vertical line in the boxplots corresponds to the median, while the diamond corresponds to the mean. The box length represents the inter-quartile range, and the whiskers extend to 1.5 times the inter-quartile range. Generalized heritability is given at the left of each boxplot. **B** Proportion of grain yield variance explained by GLAI QTLs. Proportion of explained variance, computed as *r*^2^, is given in percent. Color use is the same as panel A. The number of GLAI QTLs with a significant effect on grain yield in the network, or each environment, is given at the right of each bar

In the GY_11_ network, maize GY measured over 11 WD trials spread over five locations in three countries and four years varied on average from 53 to 89 q.ha^−1^ with a generalized heritability between 0.63 and 0.80, which underlines the diversity of drought stress that occurred during these trials (Fig. 6A). The use of a multi-environment backward selection model from all GLAI QTLs (120 unique regions for D_V_, D_S_, AUC_V_, AUC_F_, AUC_S_, GLAI_M_) led to the identification of 16 GLAI QTLs distributed over seven chromosomes having a significant effect on GY in this drought stressed network (Fig. 6B, Fig. S9). Taken together, the 16 QTLs explained almost one-fifth (*r*^2^ = 0.18, *p*value < 10^–3^, Fig. 6B) of the phenotypic variability observed over the 11 environments, and from 7 to 18% of the phenotypic variability observed in each environment, with 3–8 significant QTLs. Interestingly, each GLAI trait was associated with at least one of the 16 QTLs selected by the backward selection model, confirming the relevance of the impact of the six GLAI traits on yield.

## Discussion

To quantify the changes in GLAI in diverse maize genotypes, a panel of 324 DH lines from a MAGIC population was phenotyped by UAV spectral imaging in four trials under optimal conditions and drought stress. Six traits derived from the GLAI dynamics were shown to capture a significant proportion of the phenotypic diversity of the panel. To identify the genetic basis for these secondary traits and their influence on yield and drought tolerance, two GWAS approaches were compared: a univariate model and a multivariate model.

### Traits governing GLAI dynamics are less complex than yield

The traits evaluated within the trial network showed a high heritability, except for ASI. The low heritability of ASI (*H*^2^ = 0.5) is consistent with the results of Cairns et al. (2013), Trachsel et al. (2016) and Bouchet et al. (2017) but much lower than the heritability reported for American and Chinese nested association mapping populations (Li et al. 2016c). The heritability of FF was also similar to previous estimates (Li et al. 2016c; Bouchet et al. 2017). Yield and its components show strong heritability compared to previous studies (Trachsel et al. 2016; Li et al. 2016a; Bouchet et al. 2017; Cerrudo et al. 2018), as do the AUC (Christopher et al. 2014; Trachsel et al. 2016; Cerrudo et al. 2018) and senescence-related traits (Messmer et al. 2011; Ziyomo and Bernardo 2013; Almeida et al. 2014; Yang et al. 2017). The new traits describing vegetative growth of the canopy (D_V_, GLAI_M_, AUC_V_) showed similar heritability to traits such as leaf number and leaf size (Bouchet et al. 2017; Pan et al. 2017; Wang et al. 2017; Zhao et al. 2019).

GLAI traits were more heritable than agronomic traits, including yield and its components, and often had higher genetic variance. Moreover, although heritability gap between GY and GLAI traits is modest when computed at the network level, it can be noted that differences increase when heritability is evaluated at the trial level with a mean (maximum) gain of heritability for GLAI traits compared to GY of 0.10 (0.15) and 0.21 (0.3) for 16STPAULWW and 16STPAULWD, respectively, and 0 (0.12), 0.17 (0.28) for 17STPAULWW and 17STPAULWD, respectively. In addition, the G × E interaction was lower for most GLAI traits than for agronomic traits (Table 1, Table S4). Bouchet et al. (2017) and Yang et al. (2014) made similar observations regarding architectural canopy traits (number of leaves, size of leaves) and yield components, with comparable values of the ratio $\sigma_{ge}^{2} /\sigma_{g}^{2}$. In our study, the heritability was slightly higher when G × E interaction ratios were lower, whereas this relationship was more pronounced in the results of Yang et al. (2014) but not evidenced in results from Bouchet et al. (2017). These differences confirm that beyond the characteristics specific to the traits analyzed, the conclusions drawn from such genetic analyses depend on the population considered and the experimental network. For this study, we used a panel produced from 16 lines representative of the genetic diversity used for hybrid production in temperate zones, encompassing a very broad diversity of canopy structures (number, size, color and angle of the leaves). While the test-cross was necessary for the results to be pertinent to elite or breeding material as correlations are often weak between lines and hybrids (Cairns et al. 2012; Trachsel et al. 2016), phenotypic evaluation after the test cross clearly reduced the panel diversity. The limited size of this experimental network probably also did not reveal all the existing variability in our panel for the considered traits. Our results suggest that the traits extracted from GLAI dynamics are less complex than yield-related traits.

### Larger green leaf area increases grain yield under optimal conditions and moderate drought

GLAI traits were highly correlated with each other at the phenotypic and genetic levels (Figs. S3 and S8). They were also significantly correlated with yield and flowering. However, yield was more correlated with GLAI than with flowering, which is consistent with the results of Trachsel et al. (2016) and Cerrudo et al. (2018). It is interesting to note that the duration of the vegetative phase (D_V_) and the duration of the senescence phase (D_S_) were negatively correlated, and that this opposition was the main factor differentiating the genotypes of the panel (Fig. 2). This relationship shows that it is possible to lengthen the senescence phase by shortening the vegetative phase while maintaining the same total duration. Thus, the duration of grain filling could possibly be increased without altering the maturity date of a genotype. Trachsel et al. (2017) obtained similar results showing under optimal and stressed conditions that, provided vegetative development was vigorous, earlier flowering extended the grain filling duration, thus increasing grain yield. In our study, the traits most consistently and strongly correlated overall with yield were those that reflect vigorous vegetative development and hence a large green leaf area during grain filling: AUC_V_, AUC_F_, GLAI_M_ and AUC_S_ with 0.14 ≤ *r*_*p*_ ≤ 0.50 and − 0.04 ≤ *r*_*g*_ ≤ 0.94. These moderate correlations were expected as the proportion of yield variation explained by individual traits is known to be low at intermediate levels of water-deficit and stress, like the ones experienced in this study (Cooper and Messina 2023).

Stay-green, defined as the maintenance of photosynthetic activity during filling, is one of the major determinants of yield under optimal and stressed conditions (Wolfe et al. 1988; Cairns et al. 2012; Almeida et al. 2014; Kante et al. 2016) and has played a major role in the improvement of new hybrids in recent decades (Duvick 2005). Stay-green may be the consequence of increased green surface area before flowering, postponement of senescence, or slower senescence (Thomas and Howarth 2000; Christopher et al. 2014, 2016). AUC_S_ is a good indicator of stay-green because it incorporates each of these aspects. Indeed, AUC_S_ was the GLAI trait most correlated with yield in our four environments (Figs. S3 and S8) which confirms the major interest of this trait. While numerous studies stated that stay-green can originate from ‘shifting water’ from pre- to post-anthesis, whether by optimal transpiration efficiency, root uptake or reduced transpiration area, that would lengthen senescence duration (Borrell et al. 2000, 2014), it is interesting to note that in this study, stay-green was the result of more green surface grown before flowering and an acceleration of senescence during the rapid phase as seen by the strong correlations between AUC_S_, GLAI_M_ and S_RS_. The weak correlation of AUC_S_ and D_S_ shows that slower senescence was not a contributory factor. Moreover, D_S_ was only weakly correlated with GY. A well-developed foliar system was therefore central to the development of yield within our experimental network whether in optimal conditions or moderate drought stress with full irrigation at the end of the cycle. Maintaining leaf growth and delaying senescence increase transpiration, but if water reserves are restored before the end of the cycle, gas exchange and photosynthesis can still be maximized (Tardieu 2012). If not, with a severe terminal stress, maintaining leaf growth would be deleterious. In such conditions, typical to the Australian environment type experienced by Borrell et al. (2000, 2014), it would be expected that GY would be negatively correlated with traits that increase leaf transpiration, such as areas under the curve or GLAI_M_, while being positively correlated with DS.

These specificities highlight that secondary traits are only of interest if associated with well-characterized types of environments, which refers to ideotyping analysis (Hammer et al. 2014; Bustos-Korts et al. 2019b; Cooper and Messina 2023). Indeed, correlation between secondary traits and GY varies between environment type, and even across the crop cycle in a given environment. With a good understanding of environment type, one can expect identifying the most relevant secondary traits that maximize correlation with GY to build one or more associated ideotype, if there is sufficient knowledge about environment variability (Cooper and Messina 2023). This will thus allow a better breeding efficiency through indirect selection.

### Multivariate GWAS is the most powerful approach

With the combination of very dense genotyping data (7,806,995 SNPs) and two GWAS approaches, many associations (167,854) were detected for 11 traits measured in four environments. The multivariate approach (M_MV_) detected many more associations, including most of the associations revealed by the univariate approach (M_UV_, Fig. S5). Moreover, M_MV_ revealed many more associations common to several traits (79% of associations) than M_UV_ and explained a greater part of the phenotypic variance, particularly for yield (Fig. 5).

The power gained in GWAS with the multivariate approach compared to the univariate approach has been demonstrated by several studies (Zhu and Zhang 2009; O’Reilly et al. 2012; Stephens 2013; Galesloot et al. 2014; Porter and O’Reilly 2017). Taking into account the covariances among traits brings additional information that increases the power in detecting genetic variants involved in the control of several traits, but also variants affecting a single trait (Ferreira and Purcell 2009; Stephens 2013; Carlson et al. 2019). However, the use of multivariate models may be limited by the number of phenotypes that can be integrated. For example, in this study, a maximum of eight traits could be integrated into the multivariate model fitted with the GEMMA software. In recent years, many tools have been developed for multi-trait GWAS that differ in terms of the number of analyzable traits, calculation times and assumptions on the distribution of phenotypes (O’Reilly et al. 2012; Sluis et al. 2013; Stephens 2013; Zhou and Stephens 2014). These tools also have a very variable power depending on the phenotypic correlations and the genetic architecture of the traits studied (Porter and O’Reilly 2017).

In this work, we used a two-step approach for its computational efficiency at the cost of a probable loss of information between the two steps. The gain in power noted in several studies using functional mapping, and especially random regression (Wu and Lin 2006; Li et al. 2010; Ning et al. 2017, 2018; Campbell et al. 2019; Moreira et al. 2020) may have better enhanced our understanding of the timing of the genetic control of GLAI dynamics in response to drought stress. However, computationally it would not have been practicable with our dense genotyping data and the backward-elimination method (Ning et al. 2017; Moreira et al. 2020).

### GLAI and agronomic traits exhibit polygenic architecture and common genetic determinants

The associations we detected overlap strongly with the genetic determinisms of GLAI traits, yield and flowering (Fig. S6). Our results, like those of Cheverud (1988), Bouchet et al. (2017), Pan et al. (2017), Sodini et al. (2018) and Yuan et al. (2019) show that correlations at the phenotypic level are good indicators of the sharing of genetic determinants (pleiotropy or genetic linkage) between two traits (Fig. S10A). This indicates that it is possible to select secondary yield traits quite efficiently based on phenotypic correlations. However, we observed only a moderate link between phenotypic and genetic correlations (Fig. S10B), which demonstrates the complementarity of these two pieces of information. This partial discrepancy is probably due to permanent environmental effects at the plot level throughout the cycle, captured by the temporal approach implemented in this study (Hadfield et al. 2007; Canela-Xandri et al. 2018).

Common genetic determinisms between earliness, plant architecture and changes in leaf area during the cycle are well known (Li et al. 2016b; Bouchet et al. 2017; Pan et al. 2017; Condorelli et al. 2018; Spindel et al. 2018). The entire foliar and reproductive apparatus can be traced back to the activity of the apical meristem and the floral meristems it initiates (Kwiatkowska 2008), which is probably the reason for these common determinisms (Thompson et al. 2015; Baute et al. 2015). The impact of changes in leaf area on light interception, gas exchange and water fluxes explains the large number of QTLs affecting both yield and leaf area dynamics under optimal or stressed conditions (Trachsel et al. 2016; Cerrudo et al. 2018; Condorelli et al. 2018). In our study, however, there is less colocalization between yield and flowering than between yield and GLAI traits, which could be the consequence of purposely reducing the flowering time range of the panel. Having limited the flowering date confounding effect, it can be assumed that a large proportion of the common QTLs between yield and GLAI traits that do not colocalize with flowering are due to direct QTL effects on the GLAI. Moreover, we showed that some GLAI QTLs detected under optimal conditions colocalize with yield QTLs detected under stressed conditions. This means yield under drought stress is partly due to GLAI QTLs detected in the absence of stress, which may facilitate the prediction of grain yield in breeding.

Individually, the detected QTLs reveal a complex architecture for each trait, with 4% of phenotypic variance explained on average, and a maximum of 11%. A polygenic architecture with many low-effect QTLs and a few medium-effect QTLs is fairly consistent with the results of Bouchet et al. (2017) and Pan et al. (2017) for leaf architecture, yield and flowering, and those of Trachsel et al. (2016) and Cerrudo et al. (2018) for normalized difference vegetation index dynamics, senescence and yield. In addition, our results show that individually, GLAI QTLs explain a larger proportion of the phenotypic variance at the network scale than yield or flowering QTLs, which is in good agreement with the high stability of the GLAI QTLs between the different environments. Similarly, the total *r*^2^ explained by all QTLs is greater for GLAI (AUC_F_, AUC_S_, GLAI_M_) than for yield (Fig. 5, M_UV_), while more QTLs were detected for yield (+ 25%). It therefore appears that the genetic architecture of GLAI is simpler than that of yield with fewer genetic factors, more stable between environments, exhibiting somewhat larger individual effects.

### GLAI dynamics is a promising secondary trait for drought tolerance

Sixteen GLAI QTLs were shown to explain GY measured in 11 environments under drought stress conditions (GY_11_). They explained a significant part of the variance of GY_11_ (18%), despite the small size of the initial experimental network in which the QTLs were detected (only four environments, two of which under optimal conditions). It is interesting to note that the 16 QTLs had much more variable effects between the 11 environments of GY_11_ (Fig. S9) than in the four relatively similar environments where GLAI data was collected (Fig. S7). GLAI QTLs detected in a specific environment are therefore informative of observed yield in a wide range of contrasting environments.

Among the 16 QTLs associated with GY_11_, five have similar effects in all environments, such as C1:57,391,598 whose allele from B73 increases grain yield. This QTL colocalizes with a QTL identified in the reference network whose B73 allele increases AUC_S_ and GLAI_M_. It also colocalizes with a stay-green QTL found by Wang et al. (2016) and two leaf-width QTLs detected by Wang et al. (2018). A gain in GY when there is more green leaf area at flowering and during grain filling is consistent with the observations made on the reference network. Another example is C5:10,876,996, whose allele from B73 leads to a yield decrease, colocalizing with a QTL whose B73 allele increases D_S_ and decreases GY under drought stress in the reference network. Consistently, Almeida et al. (2014) previously detected three QTLs affecting chlorophyll content during grain filling that also colocalize with C5:10,876,996. However, as observed by Trachsel et al. (2017), one would expect that increasing the duration of filling would lead to a yield increase, at least in environments without severe terminal stress. But, as D_S_ is negatively correlated with D_V_, AUC_V_, and GLAI_M_, the decreased vigor of vegetative development might explain the yield loss (Fig. S3). Finally, QTL C3:19,131,545 is typical of QTLs with unstable effects between environments. Indeed, the reference allele of this QTL has the strongest GY effect detected in this study, varying from − 6.9 to + 3.1 q.ha^−1^ (− 14.4 to + 4.8% of the mean yield) for 17SZEGEDWD and 16BLOISWD, respectively. It colocalizes with QTLs whose reference allele increases FF, AUC_V_, AUC_S_, and/or GLAI_M_ under optimal and stressed conditions and GY under optimal conditions in the reference network. This QTL therefore reveals the variable effect of a developed leaf area over the entire cycle. While both environments experienced drought stress during flowering and grain filling, it appears that 17SZEGEDWD where B73 allele has the strongest deleterious effect on GY, was much warmer than 16BLOISWD where B73 allele increases GY (23 °C versus 18 °C in average between flowering and harvest). It would therefore seem that thermal stress coupled with drought stress is the reason for the negative effect of a more developed foliar area on GY, possibly cause by increased evapotranspiration induced by the rise in temperature. The interpretations proposed here for these three QTLs remain tentative, because although GLAI traits show little G × E interaction in the reference network, it is possible that the effect of these QTLs on GLAI changes direction within the nine additional environments considered here.

All these 16 QTL colocalize with previously identified QTL or meta-QTL for leaf architecture, plant architecture and/or GY and GY components (Trachsel et al. 2016; Zhang et al. 2016, 2017; Chen et al. 2017; Zhao et al. 2018). Validated on this 11 WD trials network, these GLAI QTL affecting GY seem interesting candidates to be further investigated.

### Perspectives and implications for breeding

In this study, we describe six new traits related to GLAI dynamics that constitutes relevant secondary traits for GY improvement under optimal and drought conditions. The other 18 GLAI traits extracted from the dynamics that were not analyzed in detail in this study show overall similar characteristics to those of the six traits analyzed (Fig. S4, Table S4). These traits could therefore be particularly interesting for new breeding programs, depending on the considered ideotype. Indeed, although the selection efficiency expected by using these traits individually can be close to that of yield alone, due to a limited gain in heritability or a genetic correlation with yield that can be reduced in certain cases, it as to be noted that using an ideotype selection index based on several of these traits can greatly increase the efficiency of indirect selection (Bänziger and Lafitte 1997; Monneveux et al. 2006; Ziyomo and Bernardo 2013). Additionally, it can be valuable to decrease selection accuracy if economic efficiency is increased in breeding programs (van Eeuwijk et al. 2019). Indeed, by selecting secondary traits such as GLAI dynamics, it is possible to quickly phenotype a large number of individuals at low cost early in the cycle, which could at least allow eliminating the individuals that are strongly divergent from the desired ideotype (Araus et al. 2018). Consistently, Bustos-Korts et al. (2019a) restated in a recent simulation study that intermediate secondary traits, such as green canopy dynamics, are particularly well suited for field evaluation during early breeding cycles to improve selection efficiency.

To improve breeding efficiency, the many QTL identified in this work could be used in a marker-assisted selection program. However, it can be noted that the most heritable traits with the least G × E interaction were not necessarily the best explained by the detected QTLs in the multivariate GWAS approach (for example, D_V_). This suggests that these traits probably have a polygenic architecture with very weak effects, as is postulated for plant height (Peiffer et al. 2014). In this context, a genomic selection approach would undoubtedly be more effective than an approach based on QTL detection to increase the efficiency of breeding programs (Spindel et al. 2015), as shown by Bouidghaghen et al. (2023) for D_V_. Indeed, the use of secondary traits in genomic selection to predict yield greatly increases the quality of prediction (Rutkoski et al. 2016; Sun et al. 2017; Crain et al. 2018; Bustos-Korts et al. 2019a). However, genomic selection should not be substitute to QTL detection, which may be more efficient in certain cases (Spindel et al. 2015; Yuan et al. 2019). Cerrudo et al. (2018) proposed an optimal approach of using the QTLs identified by GWAS in the first stages of a breeding program to enrich the material with strong-effect alleles, then using genomic selection to improve grain yield by taking advantage of low-effect loci. Alternatively, the integration of previously detected QTLs into a genomic prediction model has also performed well, provided the QTLs explain more than 10% of the variance (Bernardo 2014; Spindel et al. 2016).

Finally, the best strategies to take advantage of these new secondary traits will only be identified by deepening our knowledge and understanding of how and when they confer adaption to a specific environment type, probably through a mixture of ecophysiological modeling and empirical validation.

### Coupling ecophysiological and genetic models to gain in knowledge, understanding and power

More and more approaches combine ecophysiological modeling and genetic models through a framework that can go from sequential to fully nested. This coupling allows all these approaches to gain knowledge, understanding and/or predictive ability depending on whether the emphasis is placed on ecophysiological or genetic modeling (Messina et al. 2023).

Our two-step approach uses a simple physiological model and high-throughput field phenotyping to extract the components of GLAI dynamics and then characterize their genetic determinism. While the main focus is on genetic modeling, the use of a physiological model has several advantages: the dynamic modeling of GLAI allows (i) to extract descriptive genotypic parameters for different physiological phases, which is highly desired since the correlation between secondary traits and GY varies during the cycle depending on the type of environment, and (ii) to increase the heritability of the extracted traits (Bustos-Korts et al. 2019a, b). The main contribution of this work is a better knowledge of the genetic determinism of GLAI dynamics and its link with GY made possible by fine field phenotyping and a computationally efficient multi-trait and multi-environment QTL approach for a large number of genotypes and a dense genotyping. Indeed, this study is one of the first to study the genetic determinism of maize GLAI temporal evolution over the whole cycle in the field. As seen in similar works (Millet et al. 2016, 2019; Touzy et al. 2019; Bouidghaghen et al. 2023), this coupled modeling approach allowed us to gain power in GWAS but also to better understand the physiological mechanisms relevant for adaptation to a particular environment through the analysis of correlations between traits and colocalization of QTL (Bustos-Korts et al. 2019b), which constitutes a first step toward ideotype design. This understanding is however limited by the limited range of environments and the relationships between evaluated traits.

Studies that put more effort into ecophysiological modeling are able to greatly expand our understanding of G × E interactions and adaptive traits for each type of environment and therefore propose one or more ideotypes when necessary (Bustos-Korts et al. 2019b; Cooper and Messina 2023; Messina et al. 2023). Today, the most advanced approaches to couple ecophysiological and genetic modeling (Technow et al. 2015; Cooper et al. 2016; Messina et al. 2018; Bustos-Korts et al. 2019b) consist of a one-step approach that integrates biological information from several environments through an ecophysiological model, directly into genomic prediction algorithms to increase the predictive power and understanding of G × E interactions in the breeding network. While they surely constitute the next step to take advantage of a better G × E understanding to improve the breeding efficiency, this kind of approaches remains complementary with approaches like our, based on targeted phenotyping in a well-characterized environment, because they allow to gain empirical knowledge which can then enrich the ecophysiological and genetic model couple (Yin et al. 2004; Bustos-Korts et al. 2019a; Hammer et al. 2019).

## Conclusion

Genetic determinants of maize canopy characteristics were found by measuring GLAI dynamics over the crop cycle in a diverse panel from a MAGIC population genotyped at a very high density. Due to the difficulty of monitoring GLAI in the field for a large number of genotypes, this study is one of the first to study the genetic determinism of its temporal evolution over the whole cycle. From the GLAI dynamics, 24 traits were derived, six of which were shown to capture a significant part of the panel diversity. Six GLAI traits correlated with yield under optimal and drought conditions are more variable, more heritable, and less subject to G × E interaction than yield, making them particularly suitable secondary traits for drought tolerance improvement. The multivariate GWAS approach explained a large proportion (40–60%) of the traits variance. The QTLs identified show a strong overlap between the genetic determinants of GLAI, GY and flowering. A subset of GLAI QTLs was validated as explaining nearly one-fifth of the GY variation measured on 11 environments under drought stress. GLAI traits are promising for the relative simplicity of their genetic architecture and medium genetic effects that are relatively stable between environments.

### Supplementary Information

Below is the link to the electronic supplementary material.

Supplementary file1 (PDF 5891 KB)

## Data Availability

Data and plant material are available on reasonable request.
