# Supplementary material for: Maize green leaf area index dynamics: genetic basis of a new secondary trait for grain yield in optimal and drought conditions
Source: Theor Appl Genet. 2024 Mar 5;137(3):68. doi: 10.1007/s00122-024-04572-6 (PMC10914915; doi:10.1007/s00122-024-04572-6)
Supplement: Supplementary file 1 — Supplementary file1 (PDF 5891 KB) [file 122_2024_4572_MOESM1_ESM.pdf]

Supplementary Material

Maize Green Leaf Area Index dynamics: genetic basis of a new secondary trait for grain yield in optimal and drought conditions

Authors

Justin Blancon[1,2], Clément Buet[1], Pierre Dubreuil[1], Marie-Hélène Tixier[1], Frédéric Baret[3], Sébastien Praud[1]

[1] Biogemma, Centre de Recherche de Chappes, 63720 Chappes, France
[2] UMR GDEC, INRAE, Université Clermont Auvergne, 63000 Clermont-Ferrand, France
[3] UMR EMMAH, UMT CAPTE, INRAE, 84914 Avignon, France
Corresponding author: Justin Blancon, ORCID 0000-0002-9780-531X, justin.blancon@inrae.fr

Fig. S1 Water balance analysis for 16STPAUL and 17STPAUL trials, in well-watered (WW) and water-deficient (WD) conditions **1**

Fig. S2 Automated pipeline for RGB and multispectral images processing **2**

Model S1 Estimation of adjusted means **3**

Eq. S1 Estimation of generalized heritabilty **3**

Model S2 Analysis of variance components **3**

Model S3 Univariate GWAS **3**

Model S4 Multivariate GWAS **3**

Model S5 QTL identification **3**

Table S1 Description of GY_{11} network **4**

Fig. S3 Phenotypic correlations between the six GLAI traits and the five agronomic traits **5**

Fig. S4 Impact of drought stress on the 18 additional GLAI traits measured on 324 maize hybrids in four enviroments **6**

Fig. S5 Distribution of GLAI traits associations between M_{UV} and M_{MV} approaches **7**

Table S2 Summary of the number of SNPs associated with traits in each of four environments in which 324 maize hybrids were evaluated **8**

Table S3 Description of the QTLs selected in each environment after backward elimination with a multienvironment multilocus model (Model S5) **9**

Fig. S6 Colocalization analysis of detected QTLs **19**

Fig. S7 QTLs Allelic effects in the four environments in which 324 maize hybrids were evaluated **24**

Fig. S8 Genetic correlations between the 6 GLAI traits, GY and FF **25**

Table S4 Variance decomposition for the 18 additional GLAI traits on the experiment network **26**

Fig. S9 Allelic effects of GLAI QTLs with significant effect on GY_{11} **27**

Fig. S10 Consistency between phenotypic link and genetic link **28**

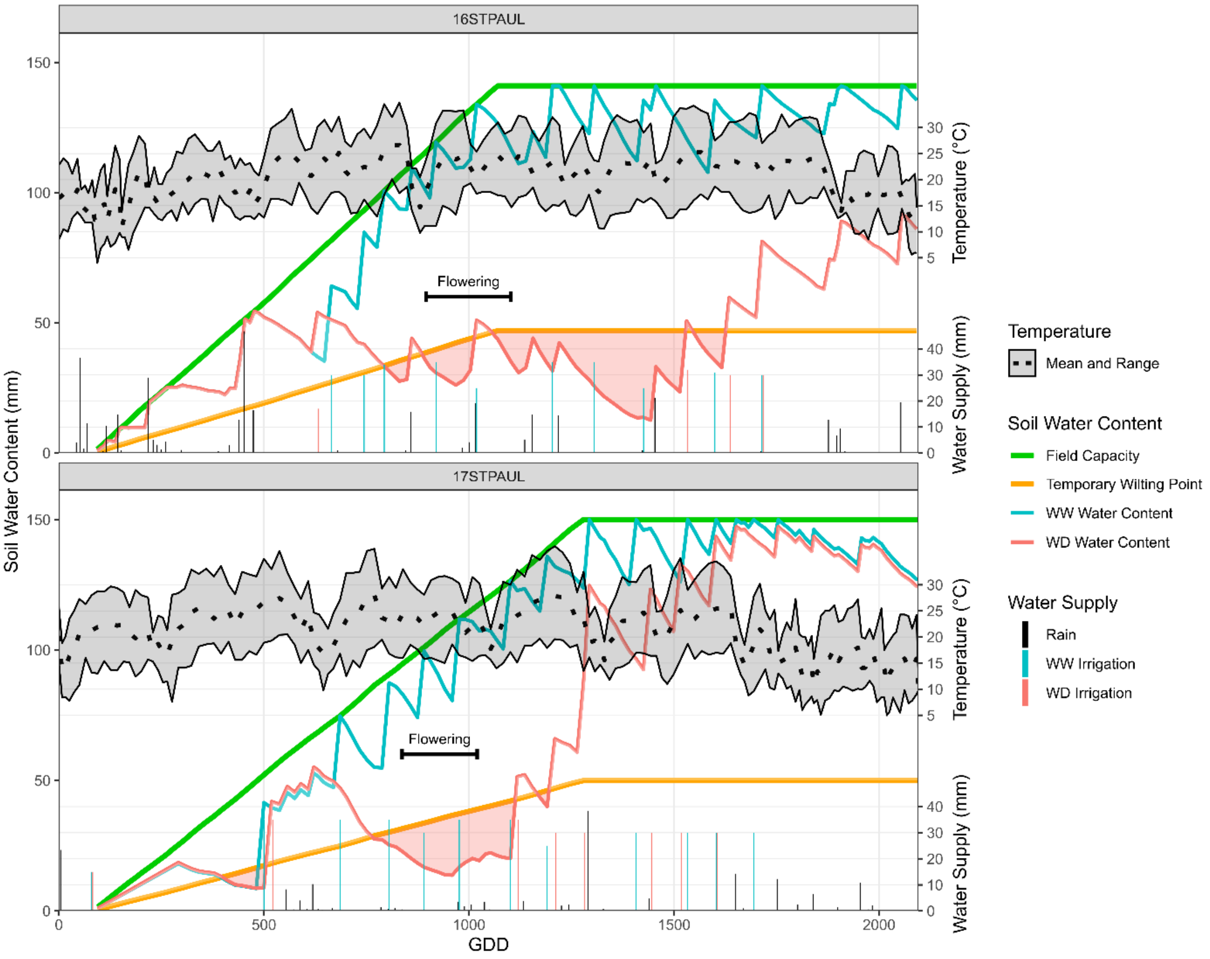

Fig. S1 Water balance analysis for 16STPAUL and 17STPAUL trials, in well-watered (WW) and water-deficient (WD) conditions. The analysis indicates if plants benefit from readily usable water reserves, between field capacity (green line) and the temporary wilting point (orange line). A water stress occurs when the water content falls below the temporary wilting point. Water stress is represented by a red area. Rainfall and irrigation are indicated with vertical bars, while the black dotted line and the grey area indicate mean temperature and temperature range respectively. Flowering ranges are indicated with black thick segment.

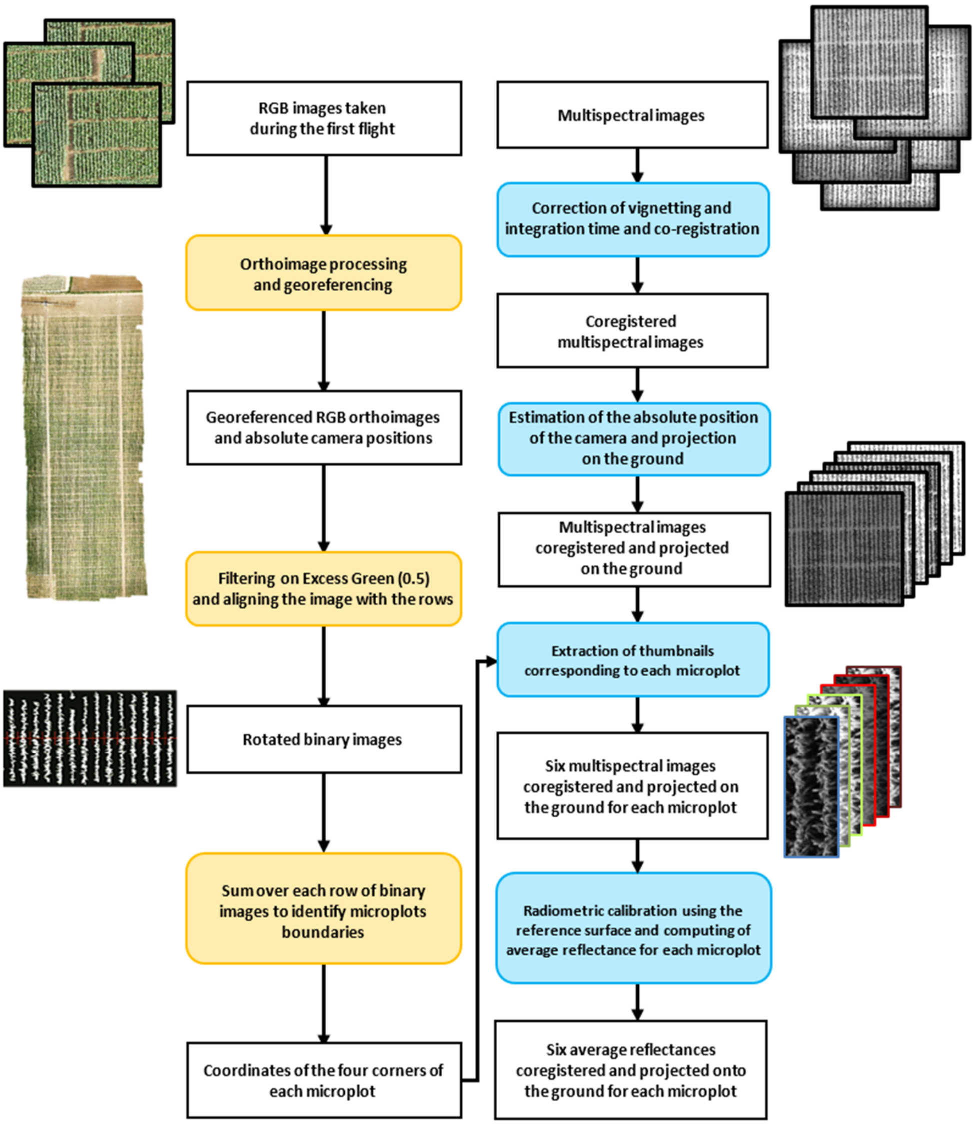

Fig. S2 Automated pipeline for RGB and multispectral images processing. On the left, the processing of RGB images consists of assembling the trial orthoimage and delimiting the microplots. On the right, the multispectral images are corrected and calibrated to extract an average reflectance value per microplot.

Model S1 Estimation of adjusted means

$$y = \mathbf{1}\mu + X\beta + W\gamma + e \quad (1)$$

where y is the phenotypic values vector, μ the overall mean, β the block fixed effect, and γ the genotype fixed effects vector, while $\mathbf{1}$ is the unit vector, X and W are the incidence matrices for the block and genotype effects respectively, and e is the residual effects vector $e \sim \mathcal{N}(0, \sigma_e^2 (R_r \otimes R_c))$ with R_r and R_c the correlation matrices for the row and column first-order autoregressive processes respectively (as proposed by Gilmour et al. 1997), and $\otimes$ the Kronecker product.

Eq. S1 Estimation of generalized heritabilty

Model S1 was fitted again, with genotype effect considered as a random to compute generalized heritability (Cullis et al. 2006):

$$H^2 = 1 - \frac{\bar{v}_{\Delta BLUP}}{2\sigma_g^2}$$

where σ_g^2 is the genetic variance and $\bar{v}_{\Delta BLUP}$ the mean variance of the difference between two best linear unbiased predictions.

Model S2 Analysis of variance components

To analyze variance components at the network scale, we used the following model:

$$y = \mathbf{1}\mu + D\omega + Z_g u_g + Z_{ge} u_{ge} + e \quad (2)$$

where $\boldsymbol{y}$ is the phenotypic values vector, $\boldsymbol{\mu}$ the overall mean, $\boldsymbol{\omega}$ the vector of other fixed effects (environment effect and nested block effect), $\boldsymbol{u_g}$ the vector of random genetic background effects, $\boldsymbol{u_{ge}}$ the vector of random GxE interaction effects, and $\boldsymbol{e}$ the vector of random residual effects; while $\mathbf{1}$ is the unit vector, $\boldsymbol{D}$, $\boldsymbol{Z_g}$ and $\boldsymbol{Z_{ge}}$ are the incidence matrices linking $\boldsymbol{y}$ to $\boldsymbol{\omega}$, $\boldsymbol{u_g}$ and $\boldsymbol{u_{ge}}$ effects with $\boldsymbol{u_g \sim \mathcal{N}(0, \sigma_g^2 I_g)}$, $\boldsymbol{u_{ge} \sim \mathcal{N}(0, \sigma_{ge}^2 I_{ge})}$ and $\boldsymbol{e \sim \mathcal{N}\left(0, \sigma_{e,k}^2 (R_{r,k} \otimes R_{c,k}) \otimes I_e, k \in [\![1,4]\!]\right)}$, where $\boldsymbol{R_{r,k}}$ denotes the correlation matrices for row and $\boldsymbol{R_{c,k}}$ for column first-order autoregressive processes in environment $\boldsymbol{k}$; $\boldsymbol{I_g}$, $\boldsymbol{I_{ge}}$, $\boldsymbol{I_e}$ are the identity matrices and $\otimes$ is the Kronecker product.

Model S3 Univariate GWAS

The association study was done trait by trait for each environment of the network, using the following univariate model:

M

$$\mathbf{M_{UV}} \qquad y = \mathbf{1}\mu + S\beta + u_g + e \quad (3)$$

where y is the adjusted means vector, μ is the overall mean, β the fixed marker effect, u_g the vector of random genetic background effects, and e the vector of residual effect, while $\mathbf{1}$ is the unit vector, S is the incidence matrix linking y to the tested marker allele and $u_g \sim \mathcal{N}(0, \sigma_g^2 K)$ and $e \sim \mathcal{N}(0, \sigma_e^2 I_e)$, where K is the LOCO Kinship matrix and I_e is the identity matrix.

Model S4 Multivariate GWAS

The general structure of **Model S3** was unchanged, but its dimensions were extended to *t* traits. For *n* genotypes, we adjusted the following multivariate model:

$$\mathbf{M_{MV}} \qquad \begin{bmatrix} y_{11} & \cdots & y_{1t} \\ \vdots & \ddots & \vdots \\ y_{n1} & \cdots & y_{nt} \end{bmatrix} = \mathbf{1}[\mu_1 \cdots \mu_t] + \begin{bmatrix} s_1 \\ \vdots \\ s_n \end{bmatrix} [\beta_1 \cdots \beta_t] + \begin{bmatrix} u_{11} & \cdots & u_{1t} \\ \vdots & \ddots & \vdots \\ u_{n1} & \cdots & u_{nt} \end{bmatrix} + \begin{bmatrix} e_{11} & \cdots & e_{1t} \\ \vdots & \ddots & \vdots \\ e_{n1} & \cdots & e_{nt} \end{bmatrix} \quad (4)$$

where $u_g \sim \mathcal{N}(0, \Sigma_g \otimes K)$ and $e \sim \mathcal{N}(0, \Sigma_e \otimes I_e)$, Σ_g and Σ_e are respectively the genetic and residual $t \times t$ heterogeneous variance-covariance matrices, K is the LOCO kinship matrix, and I_e is the identity matrix.

Model S5 QTL identification

We used a multienvironment multilocus backward selection model to identify SNP clusters playing a major role in the genetic determinism of each trait in the trial network.

$$y = \mathbf{1}\mu + X\beta + u_g + e \quad (5)$$

where β is the $(m \times 4)$ matrix of environment specific effect of the m considered clusters, and X is the genotyping matrix linking y to β, while $u_g \sim \mathcal{N}(0, \Sigma_g \otimes K)$ and $e \sim \mathcal{N}(0, \Sigma_e \otimes I_e)$ with Σ_g as the genetic matrix of variance-covariance between environments from a first-order factor analytic model where $\Sigma_g = \Gamma\Gamma' + \phi$ with Γ a (4×1) matrix and ϕ a heterogeneous diagonal matrix, and K is the global kinship matrix, Σ_e the heterogeneous diagonal matrix of environmental residual variances, and I_e the identity matrix.

Table S1 Description of GY_{11} network. Temp, mean temperature (°C); VPD, mean vapor pressure deficit (kPa); Rad, total radiation ($MJ.m^{-2}$); Rain, rainfall (mm), Irr, irrigation (mm), GY ($q.ha^{-1}$), mean grain yield; HGM, mean grain moisture at harvest (%); KN, mean kernel number per square meter; TKW, mean thousand kernel weight (g); FF, mean female flowering date (GDD6).

Environment	Year	Country	Location	Coordinates	Design	Lines	Replicates	Temp	VPD	Rad	Rain	Irr	GY	HGM	KN	TKW	FF
14BLOISWD	2014	France	Blois	47°35′38″N, 1°19′41″E	alpha-lattice	362	2	16.7	0.78	3501	385	0	82	32	3215	256	944
15GRANEROSWD	2015	Chile	Graneros	34°03′53″S, 70°43′35″W	alpha-lattice	357	2	20.2	1.56	4116	42	624	54	12	2216	246	918
15BLOISWD	2015	France	Blois	47°35′38″N, 1°19′41″E	alpha-lattice	346	2	16.5	0.91	3635	255	170	69	37	2481	279	1006
15STPAULWD	2015	France	Saint-Paul-lès-Romans	45°04′06″N, 5°08′01″E	alpha-lattice	325	2	19.1	1.13	2601	528	235	63	26	2110	299	987
16BLOISWD	2016	France	Blois	47°35′38″N, 1°19′41″E	alpha-lattice	358	2	17	0.97	3336	309	85	65	33	3980	164	1138
16NERACWD	2016	France	Nérac	44°08′53″N, 0°20′42″E	alpha-lattice	358	2	19.2	0.91	2985	176	150	53	16	2044	261	936
16STPAULWD	2016	France	Saint-Paul-lès-Romans	45°04′06″N, 5°08′01″E	alpha-lattice	360	2	19.6	1.08	2969	358	109	58	23	2796	209	1010
17BLOISWD	2017	France	Blois	47°35′38″N, 1°19′41″E	alpha-lattice	350	2	17	0.89	3858	292	67	88	32	4661	192	996
17NERACWD	2017	France	Nérac	44°08′53″N, 0°20′42″E	alpha-lattice	336	2	19.7	1	2833	298	90	69	20	2553	272	934
17STPAULWD	2017	France	Saint-Paul-lès-Romans	45°04′06″N, 5°08′01″E	alpha-lattice	347	2	20.3	1.28	2990	170	235	73	23	2565	285	943
17SZEGEDWD	2017	Hungary	Szeged	46°15′18″N, 20°08′42″E	alpha-lattice	370	2	20.1	1.21	3103	199	0	54	10	2273	236	922

A

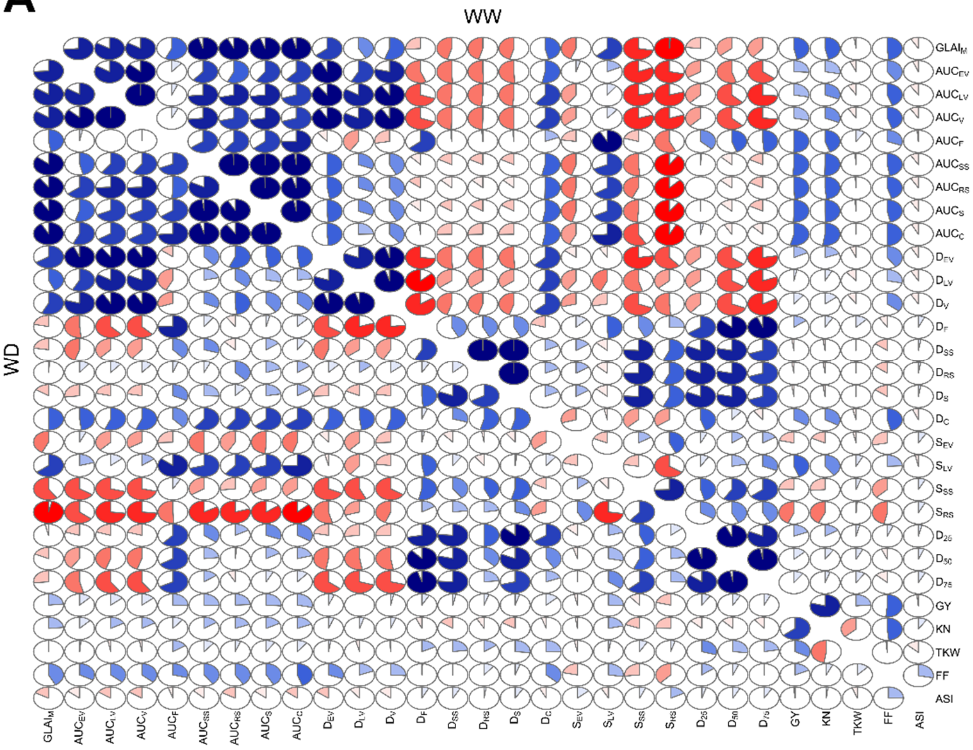

B

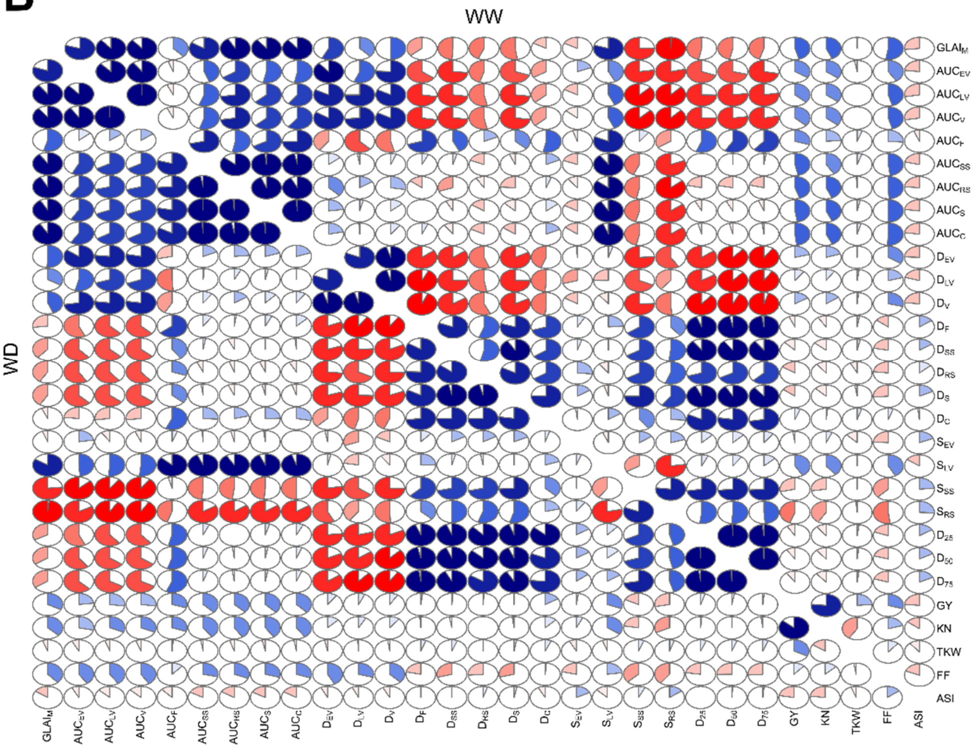

Fig. S3 Phenotypic correlations between the 24 GLAI traits and the five agronomic traits. (**A**) 16STPAUL, (**B**) 17STPAUL. The upper triangle presents the Pearson correlation coefficient between each pair of traits in the well-watered (WW) condition, with a blue pie for positive value and a red pie for a negative value. Similarly, the lower triangle presents the correlations in the water-deficient (WD) condition.

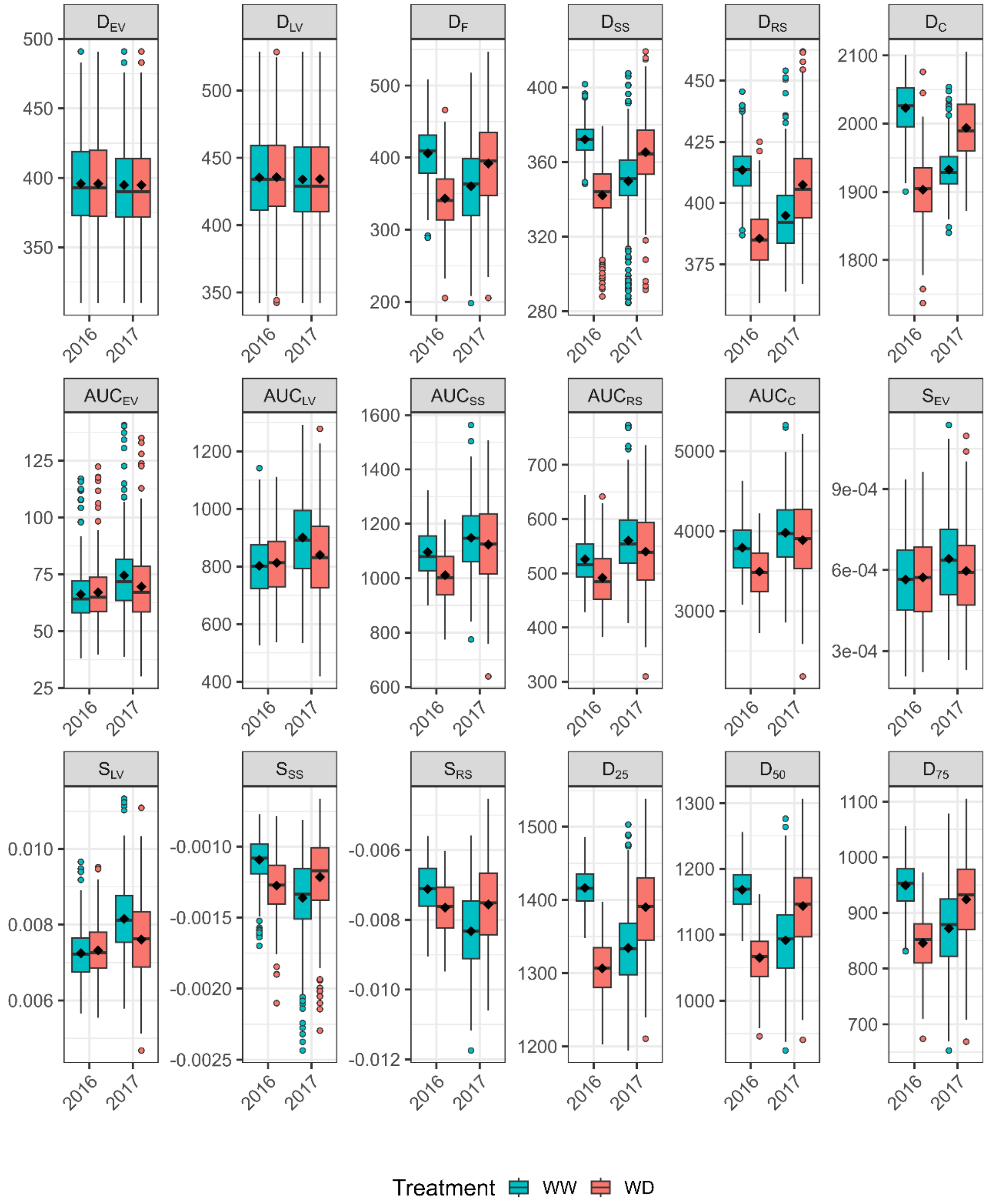

Fig. S4 Impact of drought stress on 18 GLAI traits measured on 324 maize hybrids in well-watered (WW) and water-deficient (WD) conditions. The GLAI traits are D_{EV} (GDD6), D_{LV} (GDD6), D_{F} (GDD6), D_{SS} (GDD6), D_{RS} (GDD6), D_{C} (GDD6), AUC_{EV} ($m^2.m^{-2}$.GDD6), AUC_{LV} ($m^2.m^{-2}$.GDD6), AUC_{SS} ($m^2.m^{-2}$.GDD6), AUC_{RS} ($m^2.m^{-2}$.GDD6), AUC_{C} ($m^2.m^{-2}$.GDD6), S_{EV} ($m^2.m^{-2}.GDD6^{-1}$), S_{LV} ($m^2.m^{-2}.GDD6^{-1}$), S_{SS} ($m^2.m^{-2}.GDD6^{-1}$), S_{RS} ($m^2.m^{-2}.GDD6^{-1}$), D_{25} (GDD6), D_{50} (GDD6), D_{75} (GDD6). The boxplots are constructed from the adjusted means. Horizontal lines in the box correspond to the medians, diamonds correspond to means, and circles indicate outliers. The box spans the interquartile range, and the whiskers correspond to 1.5 times the inter-quartile range.

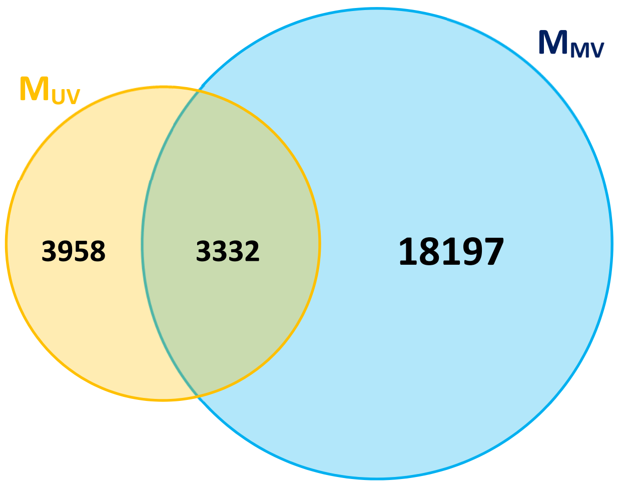

Fig. S5 Distribution of GLAI traits associations found by the univariate approach (M_{UV}) and the multivariate approach (M_{MV}). Here an association is one SNP significantly associated with a GLAI trait in a given environment.

Table S2 Summary of the number of SNPs associated with traits in each of four environments in which 324 maize hybrids were evaluated. For each maize chromosome, the number of SNP clusters and the number of QTLs retained (after the multi-environment backward elimination) are given separated by a dash. No association was found for ASI.

		SNP	Chr1	Chr2	Chr3	Chr4	Chr5	Chr6	Chr7	Chr8	Chr9	Chr10	Total
D_V	16STPAULWW	941	5-3	0-0	6-2	2-2	0-0	1-2	2-0	5-1	1-2	5-0	27-12
	16STPAULWD	1238	1-3	0-0	8-2	0-2	0-1	4-2	0-0	9-1	0-2	1-0	23-13
	17STPAULWW	1169	1-4	0-0	15-2	2-2	4-1	0-2	1-0	9-1	1-2	2-1	35-15
	17STPAULWD	3724	11-4	0-0	8-2	1-2	8-1	0-2	1-0	11-1	3-2	3-1	46-15
D_S	16STPAULWW	321	5-4	0-0	2-0	4-2	2-1	2-1	0-0	1-0	0-1	2-1	18-10
	16STPAULWD	272	4-3	0-0	0-0	0-0	1-2	0-0	0-0	4-1	0-0	0-0	9-6
	17STPAULWW	1711	0-1	0-0	9-1	4-2	2-2	0-0	0-0	4-1	1-1	0-0	20-8
	17STPAULWD	2232	12-2	0-0	14-1	1-2	8-2	0-1	0-0	8-1	7-1	3-1	53-11
$GLAI_M$	16STPAULWW	4825	3-2	5-1	12-2	8-4	2-2	0-1	0-0	9-2	2-1	3-0	44-15
	16STPAULWD	7135	2-1	1-1	5-3	3-4	0-2	2-2	1-0	10-1	6-2	0-0	30-16
	17STPAULWW	7093	3-4	3-1	7-3	5-3	2-1	2-2	2-1	7-1	2-4	2-0	35-20
	17STPAULWD	8134	16-3	4-1	17-3	1-1	17-2	0-0	1-0	14-1	6-3	2-0	78-14
AUC_V	16STPAULWW	3328	1-2	0-0	10-3	5-3	2-1	1-1	0-0	7-1	2-3	0-0	28-14
	16STPAULWD	3119	1-1	0-0	4-3	1-2	0-1	4-2	0-0	11-2	0-2	1-0	22-13
	17STPAULWW	4195	3-3	2-0	10-2	2-1	1-0	0-1	3-2	5-1	1-3	0-0	27-13
	17STPAULWD	5987	12-3	0-0	14-4	2-0	12-1	0-0	1-0	12-4	5-2	1-0	59-14
AUC_F	16STPAULWW	8144	7-2	13-2	12-6	2-0	1-1	3-0	1-0	6-2	2-1	11-1	58-15
	16STPAULWD	3770	3-3	0-2	6-3	4-1	0-2	0-0	1-1	6-0	1-1	2-1	23-14
	17STPAULWW	1713	6-4	5-2	14-4	3-1	5-2	0-0	0-0	4-1	0-1	5-0	42-15
	17STPAULWD	2695	12-4	4-1	14-4	1-1	13-2	1-0	0-0	8-2	3-0	2-0	58-14
AUC_S	16STPAULWW	4756	3-2	3-0	13-4	6-2	2-3	0-0	0-0	9-1	2-1	7-0	45-13
	16STPAULWD	6600	3-1	2-1	5-3	4-0	0-2	3-2	1-0	11-1	4-1	2-0	35-11
	17STPAULWW	7031	6-3	4-1	8-5	6-0	0-3	0-0	2-1	10-1	3-1	2-0	41-15
	17STPAULWD	8024	18-4	4-1	20-5	1-0	16-4	1-0	1-0	14-4	5-1	2-0	82-19
GY	16STPAULWW	6629	9-2	0-0	14-3	8-2	4-5	0-0	1-1	8-4	1-0	10-3	55-20
	16STPAULWD	875	3-3	1-0	8-0	5-3	1-5	0-0	1-1	1-1	0-0	1-1	21-14
	17STPAULWW	664	3-2	0-0	10-2	7-3	5-5	2-1	4-1	10-4	2-0	1-0	44-18
	17STPAULWD	2079	20-4	0-0	16-3	2-1	24-4	2-2	5-2	14-1	8-1	5-1	96-19
KN	16STPAULWW	4285	1-1	1-1	9-2	3-1	0-1	3-2	1-1	1-1	0-0	0-0	19-10
	16STPAULWD	0	0-1	0-0	0-0	0-0	0-1	0-1	0-0	0-0	0-0	0-0	0-3
	17STPAULWW	0	0-0	0-0	0-1	0-0	0-1	0-2	0-0	0-0	0-0	0-0	0-4
	17STPAULWD	102	0-0	0-0	0-1	0-0	6-2	0-0	0-0	0-0	0-0	0-0	6-3
TKW	16STPAULWW	500	0-0	0-0	0-0	3-2	0-0	0-0	0-0	1-1	0-0	0-0	4-3
	16STPAULWD	0	0-0	0-0	0-0	0-1	0-0	0-0	0-0	0-0	0-0	0-0	0-1
	17STPAULWW	0	0-0	0-0	0-0	0-2	0-0	0-0	0-0	0-0	0-0	0-0	0-2
	17STPAULWD	0	0-0	0-0	0-0	0-2	0-0	0-0	0-0	0-0	0-0	0-0	0-2
FF	16STPAULWW	4702	4-0	2-0	12-2	2-1	0-1	3-0	0-1	10-2	2-1	9-0	44-8
	16STPAULWD	6851	3-1	0-0	5-2	2-2	0-1	0-0	0-1	24-2	1-0	0-0	35-9
	17STPAULWW	12188	1-2	8-1	6-3	2-2	0-2	1-0	3-2	15-3	5-2	3-3	44-20
	17STPAULWD	25591	2-0	1-0	10-5	4-0	11-2	11-1	5-1	5-1	10-3	7-1	66-14

Table S3 Description of the QTLs in each environment after backward elimination with a multienvironment multilocus model (Model 6). The QTL boundaries were defined by adding or subtracting the extent of the linkage disequilibrium at the boundaries of the clusters that were kept after the backward elimination procedure. The QTL position is the position of the SNP with the lowest *pvalue* on the AGPv4 release of the maize genome. The effect of the QTLs comes from **Model 6**. The r^2 of a QTL corresponds to the squared Pearson correlation coefficient of the regression between the vector of effect of the SNP in the environment considered and the vector of phenotypic values. QTLs with a significant global effect in the backward multi-environment model, but that did not have any *a posteriori* significant environment-specific effect, are not reported in this table.

(**A**) D_V: Duration of the vegetative phase

Trait	Environment	Mean	Chr	SNP	Position	Start	End	Effect	r^2
D_V	16STPAULWW	797	1	C1:P206651010	206651010	206537635	206764385	-12.4	0.044
D_V	16STPAULWW	797	1	C1:P287568465	287568465	287435993	287700937	13.7	0.026
D_V	16STPAULWW	797	1	C1:P301481582	301481582	301435947	301555838	12.1	0.033
D_V	16STPAULWW	797	3	C3:P3819905	3819905	3797669	3841634	-15.5	0.052
D_V	16STPAULWW	797	3	C3:P179265374	179265374	179184075	179346673	13.0	0.018
D_V	16STPAULWW	797	4	C4:P22369755	22369755	22094727	22644783	15.2	0.044
D_V	16STPAULWW	797	4	C4:P183366399	183366399	182190908	183857724	-24.5	0.048
D_V	16STPAULWW	797	6	C6:P168463032	168463032	168446826	168479238	-9.7	0.027
D_V	16STPAULWW	797	6	C6:P168948444	168948444	168932588	168964300	-9.3	0.032
D_V	16STPAULWW	797	8	C8:P148427245	148427245	146449491	148670474	12.4	0.017
D_V	16STPAULWW	797	9	C9:P154487358	154487358	154437194	154573510	10.9	0.050
D_V	16STPAULWW	797	9	C9:P157172408	157172408	157163970	157180846	11.9	0.040
D_V	16STPAULWD	797	1	C1:P206651010	206651010	206537635	206764385	-11.9	0.043
D_V	16STPAULWD	797	1	C1:P287568465	287568465	287435993	287700937	13.2	0.025
D_V	16STPAULWD	797	1	C1:P301481582	301481582	301435947	301555838	13.1	0.036
D_V	16STPAULWD	797	3	C3:P3819905	3819905	3797669	3841634	-14.3	0.047
D_V	16STPAULWD	797	3	C3:P179265374	179265374	179184075	179346673	14.1	0.022
D_V	16STPAULWD	797	4	C4:P22369755	22369755	22094727	22644783	14.0	0.035
D_V	16STPAULWD	797	4	C4:P183366399	183366399	182190908	183857724	-22.2	0.040
D_V	16STPAULWD	797	5	C5:P188155512	188155512	188114062	188196962	12.8	0.039
D_V	16STPAULWD	797	6	C6:P168463032	168463032	168446826	168479238	-12.6	0.045
D_V	16STPAULWD	797	6	C6:P168948444	168948444	168932588	168964300	-10.5	0.043
D_V	16STPAULWD	797	8	C8:P148427245	148427245	146449491	148670474	13.8	0.021
D_V	16STPAULWD	797	9	C9:P154487358	154487358	154437194	154573510	9.9	0.044
D_V	16STPAULWD	797	9	C9:P157172408	157172408	157163970	157180846	11.0	0.034
D_V	17STPAULWW	783	1	C1:P181910488	181910488	181725538	182095438	-10.1	0.021
D_V	17STPAULWW	783	1	C1:P206651010	206651010	206537635	206764385	-14.1	0.050
D_V	17STPAULWW	783	1	C1:P287568465	287568465	287435993	287700937	13.1	0.024
D_V	17STPAULWW	783	1	C1:P301481582	301481582	301435947	301555838	13.6	0.036
D_V	17STPAULWW	783	3	C3:P3819905	3819905	3797669	3841634	-14.7	0.046
D_V	17STPAULWW	783	3	C3:P179265374	179265374	179184075	179346673	12.7	0.016
D_V	17STPAULWW	783	4	C4:P22369755	22369755	22094727	22644783	14.4	0.038
D_V	17STPAULWW	783	4	C4:P183366399	183366399	182190908	183857724	-15.6	0.020
D_V	17STPAULWW	783	5	C5:P188155512	188155512	188114062	188196962	13.6	0.040
D_V	17STPAULWW	783	6	C6:P168463032	168463032	168446826	168479238	-10.2	0.032
D_V	17STPAULWW	783	6	C6:P168948444	168948444	168932588	168964300	-9.4	0.034
D_V	17STPAULWW	783	8	C8:P148427245	148427245	146449491	148670474	14.5	0.023
D_V	17STPAULWW	783	9	C9:P154487358	154487358	154437194	154573510	11.2	0.052
D_V	17STPAULWW	783	9	C9:P157172408	157172408	157163970	157180846	10.3	0.033
D_V	17STPAULWW	783	10	C10:P22090389	22090389	21281704	22899074	12.4	0.020
D_V	17STPAULWD	784	1	C1:P181910488	181910488	181725538	182095438	-10.2	0.022
D_V	17STPAULWD	784	1	C1:P206651010	206651010	206537635	206764385	-13.8	0.049
D_V	17STPAULWD	784	1	C1:P287568465	287568465	287435993	287700937	13.3	0.024
D_V	17STPAULWD	784	1	C1:P301481582	301481582	301435947	301555838	13.8	0.036
D_V	17STPAULWD	784	3	C3:P3819905	3819905	3797669	3841634	-14.6	0.046

D_V	17STPAULWD	784	3	C3:P179265374	179265374	179184075	179346673	12.9	0.016
D_V	17STPAULWD	784	4	C4:P22369755	22369755	22094727	22644783	14.2	0.037
D_V	17STPAULWD	784	4	C4:P183366399	183366399	182190908	183857724	-15.8	0.019
D_V	17STPAULWD	784	5	C5:P188155512	188155512	188114062	188196962	14.5	0.041
D_V	17STPAULWD	784	6	C6:P168463032	168463032	168446826	168479238	-10.1	0.032
D_V	17STPAULWD	784	6	C6:P168948444	168948444	168932588	168964300	-9.6	0.035
D_V	17STPAULWD	784	8	C8:P148427245	148427245	146449491	148670474	13.9	0.021
D_V	17STPAULWD	784	9	C9:P154487358	154487358	154437194	154573510	11.2	0.053
D_V	17STPAULWD	784	9	C9:P157172408	157172408	157163970	157180846	10.4	0.034
D_V	17STPAULWD	784	10	C10:P22090389	22090389	21281704	22899074	12.5	0.020

(**B**) D_S: Duration of the senescence phase

Trait	Environment	Mean	Chr	SNP	Position	Start	End	Effect	r²
D_S	16STPAULWW	802	1	C1:P31094078	31094078	31003673	34083784	5.66	0.041
D_S	16STPAULWW	802	1	C1:P110696135	110696135	108685880	112706390	4.71	0.042
D_S	16STPAULWW	802	1	C1:P176537716	176537716	171920205	177326395	-4.55	0.024
D_S	16STPAULWW	802	1	C1:P259903749	259903749	259622364	260185134	2.58	0.022
D_S	16STPAULWW	802	4	C4:P22369755	22369755	22094727	22644783	-3.94	0.024
D_S	16STPAULWW	802	4	C4:P77205734	77205734	69166984	84191971	-8.64	0.030
D_S	16STPAULWW	802	5	C5:P3897375	3897375	3888640	3909325	-3.86	0.021
D_S	16STPAULWW	802	6	C6:P154044851	154044851	153996111	154094634	-7.31	0.040
D_S	16STPAULWW	802	9	C9:P154487358	154487358	154437194	154573510	-2.98	0.027
D_S	16STPAULWW	802	10	C10:P84121394	84121394	83009005	84814285	4.15	0.051
D_S	16STPAULWD	744	1	C1:P35374991	35374991	35277668	36837997	-4.29	0.027
D_S	16STPAULWD	744	1	C1:P110696135	110696135	108685880	112706390	3.63	0.014
D_S	16STPAULWD	744	1	C1:P287925166	287925166	287799344	288050988	-7.09	0.021
D_S	16STPAULWD	744	5	C5:P10876996	10876996	10862820	11035515	3.26	0.039
D_S	16STPAULWD	744	5	C5:P188155512	188155512	188114062	188196962	-3.31	0.024
D_S	16STPAULWD	744	8	C8:P20725203	20725203	20674880	20775526	5.88	0.041
D_S	17STPAULWW	776	1	C1:P176537716	176537716	171920205	177326395	-10.14	0.050
D_S	17STPAULWW	776	3	C3:P3819905	3819905	3797669	3841634	8.57	0.061
D_S	17STPAULWW	776	4	C4:P22369755	22369755	22094727	22644783	-8.02	0.038
D_S	17STPAULWW	776	4	C4:P77205734	77205734	69166984	84191971	-16.26	0.058
D_S	17STPAULWW	776	5	C5:P3897375	3897375	3888640	3909325	-5.60	0.011
D_S	17STPAULWW	776	5	C5:P188155512	188155512	188114062	188196962	-5.82	0.064
D_S	17STPAULWW	776	8	C8:P142785044	142785044	142504074	143243744	-5.81	0.047
D_S	17STPAULWW	776	9	C9:P154487358	154487358	154437194	154573510	-7.31	0.050
D_S	17STPAULWD	803	1	C1:P176537716	176537716	171920205	177326395	-8.91	0.033
D_S	17STPAULWD	803	1	C1:P259903749	259903749	259622364	260185134	5.38	0.027
D_S	17STPAULWD	803	3	C3:P3819905	3819905	3797669	3841634	7.45	0.038
D_S	17STPAULWD	803	4	C4:P22369755	22369755	22094727	22644783	-9.23	0.049
D_S	17STPAULWD	803	4	C4:P77205734	77205734	69166984	84191971	-12.54	0.021
D_S	17STPAULWD	803	5	C5:P3897375	3897375	3888640	3909325	-7.81	0.024
D_S	17STPAULWD	803	5	C5:P168106675	168106675	167795025	168176938	-6.54	0.035
D_S	17STPAULWD	803	6	C6:P154044851	154044851	153996111	154094634	-8.66	0.014
D_S	17STPAULWD	803	8	C8:P142785044	142785044	142504074	143243744	-7.29	0.047
D_S	17STPAULWD	803	9	C9:P154487358	154487358	154437194	154573510	-8.23	0.048
D_S	17STPAULWD	803	10	C10:P142295385	142295385	142256672	142780053	-6.53	0.043

(**C**) $GLAI_M$: Maximum GLAI

Trait	Environment	Mean	Chr	SNP	Position	Start	End	Effect	r²
$GLAI_M$	16STPAULWW	3.38	1	C1:P171257061	171257061	170800348	172285866	0.075	0.027
$GLAI_M$	16STPAULWW	3.38	1	C1:P276935262	276935262	276490206	277380388	-0.081	0.046
$GLAI_M$	16STPAULWW	3.38	2	C2:P23201978	23201978	23153799	23250729	0.035	0.032
$GLAI_M$	16STPAULWW	3.38	3	C3:P3819905	3819905	3797669	3841634	-0.051	0.040

$GLAI_M$	16STPAULWW	3.38	3	C3:P19385721	19385721	16362424	24581733	0.107	0.066
$GLAI_M$	16STPAULWW	3.38	4	C4:P1520603	1520603	1454289	2555593	-0.116	0.026
$GLAI_M$	16STPAULWW	3.38	4	C4:P18518806	18518806	18320663	18716949	-0.051	0.033
$GLAI_M$	16STPAULWW	3.38	4	C4:P189987121	189987121	189793789	190180453	-0.102	0.054
$GLAI_M$	16STPAULWW	3.38	4	C4:P221789526	221789526	217491565	223334500	0.047	0.044
$GLAI_M$	16STPAULWW	3.38	5	C5:P41052037	41052037	40400420	41702652	0.048	0.025
$GLAI_M$	16STPAULWW	3.38	5	C5:P177879071	177879071	177841968	177915807	0.053	0.034
$GLAI_M$	16STPAULWW	3.38	6	C6:P40926244	40926244	24955624	56896864	0.046	0.021
$GLAI_M$	16STPAULWW	3.38	8	C8:P127880738	127880738	121631062	130317984	0.054	0.079
$GLAI_M$	16STPAULWW	3.38	8	C8:P149450033	149450033	149174631	149725435	0.070	0.031
$GLAI_M$	16STPAULWW	3.38	9	C9:P156889489	156889489	156840311	156899900	0.043	0.035
$GLAI_M$	16STPAULWD	3.41	1	C1:P276935262	276935262	276490206	277380388	-0.072	0.043
$GLAI_M$	16STPAULWD	3.41	2	C2:P23201978	23201978	23153799	23250729	0.046	0.060
$GLAI_M$	16STPAULWD	3.41	3	C3:P3819905	3819905	3797669	3841634	-0.046	0.034
$GLAI_M$	16STPAULWD	3.41	3	C3:P19385721	19385721	16362424	24581733	0.109	0.065
$GLAI_M$	16STPAULWD	3.41	3	C3:P178236331	178236331	167465852	179220035	0.076	0.076
$GLAI_M$	16STPAULWD	3.41	4	C4:P1520603	1520603	1454289	2555593	-0.117	0.023
$GLAI_M$	16STPAULWD	3.41	4	C4:P189987121	189987121	189793789	190180453	-0.080	0.030
$GLAI_M$	16STPAULWD	3.41	4	C4:P221789526	221789526	217491565	223334500	0.048	0.038
$GLAI_M$	16STPAULWD	3.41	4	C4:P245200284	245200284	244768961	245243661	-0.061	0.029
$GLAI_M$	16STPAULWD	3.41	5	C5:P41052037	41052037	40400420	41702652	0.045	0.026
$GLAI_M$	16STPAULWD	3.41	5	C5:P177879071	177879071	177841968	177915807	0.039	0.027
$GLAI_M$	16STPAULWD	3.41	6	C6:P40926244	40926244	24955624	56896864	0.043	0.018
$GLAI_M$	16STPAULWD	3.41	6	C6:P168948444	168948444	168932588	168964300	-0.049	0.031
$GLAI_M$	16STPAULWD	3.41	8	C8:P127880738	127880738	121631062	130317984	0.052	0.073
$GLAI_M$	16STPAULWD	3.41	9	C9:P126828222	126828222	126624069	127032375	0.039	0.028
$GLAI_M$	16STPAULWD	3.41	9	C9:P156889489	156889489	156840311	156899900	0.063	0.058
$GLAI_M$	17STPAULWW	3.48	1	C1:P17974881	17974881	17904876	18048133	-0.074	0.040
$GLAI_M$	17STPAULWW	3.48	1	C1:P45444623	45444623	45338853	46123588	0.116	0.049
$GLAI_M$	17STPAULWW	3.48	1	C1:P201191429	201191429	201106138	201276815	0.069	0.021
$GLAI_M$	17STPAULWW	3.48	1	C1:P276935262	276935262	276490206	277380388	-0.069	0.024
$GLAI_M$	17STPAULWW	3.48	2	C2:P41026349	41026349	40819680	41233018	0.075	0.020
$GLAI_M$	17STPAULWW	3.48	3	C3:P3819905	3819905	3797669	3841634	-0.061	0.032
$GLAI_M$	17STPAULWW	3.48	3	C3:P11580838	11580838	11521986	11641644	-0.058	0.029
$GLAI_M$	17STPAULWW	3.48	3	C3:P19385721	19385721	16362424	24581733	0.169	0.062
$GLAI_M$	17STPAULWW	3.48	4	C4:P1520603	1520603	1454289	2555593	-0.133	0.024
$GLAI_M$	17STPAULWW	3.48	4	C4:P221789526	221789526	217491565	223334500	0.056	0.027
$GLAI_M$	17STPAULWW	3.48	4	C4:P245200284	245200284	244768961	245243661	-0.097	0.034
$GLAI_M$	17STPAULWW	3.48	5	C5:P177879071	177879071	177841968	177915807	0.068	0.048
$GLAI_M$	17STPAULWW	3.48	6	C6:P40926244	40926244	24955624	56896864	0.087	0.049
$GLAI_M$	17STPAULWW	3.48	6	C6:P168948444	168948444	168932588	168964300	-0.053	0.013
$GLAI_M$	17STPAULWW	3.48	7	C7:P176278691	176278691	176216146	176341236	-0.057	0.042
$GLAI_M$	17STPAULWW	3.48	8	C8:P127880738	127880738	121631062	130317984	0.085	0.089
$GLAI_M$	17STPAULWW	3.48	9	C9:P21726094	21726094	21618509	21833679	-0.058	0.027
$GLAI_M$	17STPAULWW	3.48	9	C9:P126828222	126828222	126624069	127032375	0.058	0.013
$GLAI_M$	17STPAULWW	3.48	9	C9:P152524629	152524629	152501340	152547918	0.064	0.074
$GLAI_M$	17STPAULWW	3.48	9	C9:P156889489	156889489	156840311	156899900	0.059	0.032
$GLAI_M$	17STPAULWD	3.37	1	C1:P17974881	17974881	17904876	18048133	-0.083	0.049
$GLAI_M$	17STPAULWD	3.37	1	C1:P45444623	45444623	45338853	46123588	0.115	0.035
$GLAI_M$	17STPAULWD	3.37	1	C1:P201191429	201191429	201106138	201276815	0.066	0.011
$GLAI_M$	17STPAULWD	3.37	2	C2:P41026349	41026349	40819680	41233018	0.099	0.027
$GLAI_M$	17STPAULWD	3.37	3	C3:P3819905	3819905	3797669	3841634	-0.051	0.023
$GLAI_M$	17STPAULWD	3.37	3	C3:P5769780	5769780	5740679	5798881	-0.105	0.032
$GLAI_M$	17STPAULWD	3.37	3	C3:P11580838	11580838	11521986	11641644	-0.056	0.027
$GLAI_M$	17STPAULWD	3.37	4	C4:P245200284	245200284	244768961	245243661	-0.094	0.033
$GLAI_M$	17STPAULWD	3.37	5	C5:P41052037	41052037	40400420	41702652	0.098	0.062
$GLAI_M$	17STPAULWD	3.37	5	C5:P177879071	177879071	177841968	177915807	0.101	0.077
$GLAI_M$	17STPAULWD	3.37	8	C8:P127880738	127880738	121631062	130317984	0.114	0.105

GLAI$_M$	17STPAULWD	3.37	9	C9:P21726094	21726094	21618509	21833679	-0.076	0.034
GLAI$_M$	17STPAULWD	3.37	9	C9:P126828222	126828222	126624069	127032375	0.084	0.022
GLAI$_M$	17STPAULWD	3.37	9	C9:P156889489	156889489	156840311	156899900	0.074	0.027

(**D**) AUC$_V$: Area under the curve during the vegetative phase

Trait	Environment	Mean	Chr	SNP	Position	Start	End	Effect	r^2
AUC$_V$	16STPAULWW	821	1	C1:P171257061	171257061	170800348	172285866	34.9	0.033
AUC$_V$	16STPAULWW	821	1	C1:P206651010	206651010	206537635	206764385	-15.6	0.020
AUC$_V$	16STPAULWW	821	3	C3:P3819905	3819905	3797669	3841634	-25.1	0.056
AUC$_V$	16STPAULWW	821	3	C3:P19131545	19131545	18590552	19616421	38.0	0.039
AUC$_V$	16STPAULWW	821	3	C3:P179265374	179265374	179184075	179346673	20.1	0.024
AUC$_V$	16STPAULWW	821	4	C4:P18518806	18518806	18320663	18716949	-16.8	0.018
AUC$_V$	16STPAULWW	821	4	C4:P22369755	22369755	22094727	22644783	21.8	0.043
AUC$_V$	16STPAULWW	821	4	C4:P183366399	183366399	182190908	183857724	-41.3	0.072
AUC$_V$	16STPAULWW	821	5	C5:P179737457	179737457	179700706	179774208	17.9	0.026
AUC$_V$	16STPAULWW	821	6	C6:P168948444	168948444	168932588	168964300	-19.7	0.029
AUC$_V$	16STPAULWW	821	8	C8:P29695849	29695849	29503920	30169775	23.1	0.010
AUC$_V$	16STPAULWW	821	9	C9:P20788638	20788638	20700310	20876966	16.7	0.018
AUC$_V$	16STPAULWW	821	9	C9:P156684586	156684586	156674600	156694572	18.7	0.035
AUC$_V$	16STPAULWW	821	9	C9:P157172408	157172408	157163970	157180846	16.6	0.035
AUC$_V$	16STPAULWD	822	1	C1:P206651010	206651010	206537635	206764385	-20.6	0.033
AUC$_V$	16STPAULWD	822	3	C3:P3819905	3819905	3797669	3841634	-23.2	0.045
AUC$_V$	16STPAULWD	822	3	C3:P19131545	19131545	18590552	19616421	41.9	0.041
AUC$_V$	16STPAULWD	822	3	C3:P179265374	179265374	179184075	179346673	24.8	0.030
AUC$_V$	16STPAULWD	822	4	C4:P22369755	22369755	22094727	22644783	17.0	0.022
AUC$_V$	16STPAULWD	822	4	C4:P183366399	183366399	182190908	183857724	-29.0	0.049
AUC$_V$	16STPAULWD	822	5	C5:P179737457	179737457	179700706	179774208	21.0	0.032
AUC$_V$	16STPAULWD	822	6	C6:P168463032	168463032	168446826	168479238	-19.9	0.043
AUC$_V$	16STPAULWD	822	6	C6:P168948444	168948444	168932588	168964300	-25.4	0.052
AUC$_V$	16STPAULWD	822	8	C8:P29695849	29695849	29503920	30169775	29.3	0.028
AUC$_V$	16STPAULWD	822	8	C8:P127880738	127880738	121409004	128734501	19.2	0.053
AUC$_V$	16STPAULWD	822	9	C9:P156684586	156684586	156674600	156694572	19.5	0.031
AUC$_V$	16STPAULWD	822	9	C9:P157172408	157172408	157163970	157180846	15.7	0.036
AUC$_V$	17STPAULWW	843	1	C1:P17974881	17974881	17904876	18044886	-22.3	0.030
AUC$_V$	17STPAULWW	843	1	C1:P171257061	171257061	170800348	172285866	32.3	0.021
AUC$_V$	17STPAULWW	843	1	C1:P206651010	206651010	206537635	206764385	-29.4	0.030
AUC$_V$	17STPAULWW	843	3	C3:P3819905	3819905	3797669	3841634	-33.7	0.045
AUC$_V$	17STPAULWW	843	3	C3:P19131545	19131545	18590552	19616421	58.1	0.045
AUC$_V$	17STPAULWW	843	4	C4:P22369755	22369755	22094727	22644783	27.4	0.030
AUC$_V$	17STPAULWW	843	6	C6:P168948444	168948444	168932588	168964300	-24.0	0.029
AUC$_V$	17STPAULWW	843	7	C7:P22679586	22679586	22230889	23033446	26.6	0.043
AUC$_V$	17STPAULWW	843	7	C7:P176278691	176278691	176216146	176341236	-21.5	0.032
AUC$_V$	17STPAULWW	843	8	C8:P127880738	127880738	121409004	128734501	30.0	0.070
AUC$_V$	17STPAULWW	843	9	C9:P20788638	20788638	20700310	20876966	28.2	0.029
AUC$_V$	17STPAULWW	843	9	C9:P152524629	152524629	152501340	152547918	24.4	0.068
AUC$_V$	17STPAULWW	843	9	C9:P157172408	157172408	157163970	157180846	22.7	0.046
AUC$_V$	17STPAULWD	812	1	C1:P17974881	17974881	17904876	18044886	-24.7	0.038
AUC$_V$	17STPAULWD	812	1	C1:P171257061	171257061	170800348	172285866	32.7	0.028
AUC$_V$	17STPAULWD	812	1	C1:P206651010	206651010	206537635	206764385	-31.7	0.035
AUC$_V$	17STPAULWD	812	3	C3:P3819905	3819905	3797669	3841634	-30.2	0.037
AUC$_V$	17STPAULWD	812	3	C3:P5769780	5769780	5740679	5798881	-36.1	0.034
AUC$_V$	17STPAULWD	812	3	C3:P19131545	19131545	18590552	19616421	47.2	0.025
AUC$_V$	17STPAULWD	812	3	C3:P179265374	179265374	179184075	179346673	24.5	0.014
AUC$_V$	17STPAULWD	812	5	C5:P177879071	177879071	177842382	177915807	21.7	0.063

AUC_V	17STPAULWD	812	8	C8:P29695849	29695849	29503920	30169775	32.2	0.012
AUC_V	17STPAULWD	812	8	C8:P77331121	77331121	77199125	77463117	28.1	0.033
AUC_V	17STPAULWD	812	8	C8:P127880738	127880738	121409004	128734501	35.7	0.089
AUC_V	17STPAULWD	812	8	C8:P149449909	149449909	149174507	149725311	33.7	0.043
AUC_V	17STPAULWD	812	9	C9:P20788638	20788638	20700310	20876966	36.0	0.043
AUC_V	17STPAULWD	812	9	C9:P157172408	157172408	157163970	157180846	28.1	0.051

(**E**) AUC_F: Area under the curve during the flowering phase

Trait	Environment	Mean	Chr	SNP	Position	Start	End	Effect	r^2
AUC_F	16STPAULWW	1355	1	C1:P182668583	182668583	182498962	182874219	34.6	0.040
AUC_F	16STPAULWW	1355	1	C1:P288692039	288692039	288572768	289743062	-26.3	0.042
AUC_F	16STPAULWW	1355	2	C2:P40568132	40568132	40348357	40787907	29.5	0.047
AUC_F	16STPAULWW	1355	2	C2:P41549595	41549595	41366255	41732944	-30.9	0.053
AUC_F	16STPAULWW	1355	3	C3:P12817456	12817456	12692687	12944381	-21.4	0.056
AUC_F	16STPAULWW	1355	3	C3:P153124599	153124599	152855739	153393459	30.0	0.071
AUC_F	16STPAULWW	1355	3	C3:P161415134	161415134	157704954	163698154	42.2	0.078
AUC_F	16STPAULWW	1355	3	C3:P163834567	163834567	163435493	164233641	-25.6	0.029
AUC_F	16STPAULWW	1355	3	C3:P168686452	168686452	168516188	168856716	-20.9	0.018
AUC_F	16STPAULWW	1355	3	C3:P170244503	170244503	170112686	170376320	55.1	0.059
AUC_F	16STPAULWW	1355	5	C5:P19149346	19149346	19116963	19181729	33.3	0.024
AUC_F	16STPAULWW	1355	8	C8:P119336307	119336307	112487560	126411262	23.8	0.027
AUC_F	16STPAULWW	1355	8	C8:P146534395	146534395	146292401	146777002	-43.0	0.062
AUC_F	16STPAULWW	1355	9	C9:P156684586	156684586	156674600	156694572	28.5	0.033
AUC_F	16STPAULWW	1355	10	C10:P149609269	149609269	149565707	149652831	22.7	0.044
AUC_F	16STPAULWD	1186	1	C1:P35943782	35943782	35850128	36037436	-22.4	0.011
AUC_F	16STPAULWD	1186	1	C1:P182668583	182668583	182498962	182874219	25.4	0.026
AUC_F	16STPAULWD	1186	1	C1:P288692039	288692039	288572768	289743062	-28.1	0.046
AUC_F	16STPAULWD	1186	2	C2:P40568132	40568132	40348357	40787907	18.4	0.025
AUC_F	16STPAULWD	1186	2	C2:P41549595	41549595	41366255	41732944	-31.8	0.052
AUC_F	16STPAULWD	1186	3	C3:P153124599	153124599	152855739	153393459	25.0	0.060
AUC_F	16STPAULWD	1186	3	C3:P161415134	161415134	157704954	163698154	27.1	0.083
AUC_F	16STPAULWD	1186	3	C3:P170244503	170244503	170112686	170376320	61.1	0.062
AUC_F	16STPAULWD	1186	4	C4:P76770266	76770266	74395495	79142729	-24.6	0.047
AUC_F	16STPAULWD	1186	5	C5:P19149346	19149346	19116963	19181729	23.3	0.010
AUC_F	16STPAULWD	1186	5	C5:P201024607	201024607	200461747	201368901	-22.7	0.022
AUC_F	16STPAULWD	1186	7	C7:P180747435	180747435	180662330	180832615	23.7	0.055
AUC_F	16STPAULWD	1186	9	C9:P156684586	156684586	156674600	156694572	20.2	0.025
AUC_F	16STPAULWD	1186	10	C10:P149609269	149609269	149565707	149652831	23.1	0.045
AUC_F	17STPAULWW	1473	1	C1:P35943782	35943782	35850128	36037436	-35.5	0.017
AUC_F	17STPAULWW	1473	1	C1:P46818021	46818021	46686790	46950585	43.5	0.025
AUC_F	17STPAULWW	1473	1	C1:P288692039	288692039	288572768	289743062	-42.2	0.045
AUC_F	17STPAULWW	1473	1	C1:P301650736	301650736	301605345	301713806	-38.8	0.051
AUC_F	17STPAULWW	1473	2	C2:P40568132	40568132	40348357	40787907	35.5	0.029
AUC_F	17STPAULWW	1473	2	C2:P41549595	41549595	41366255	41732944	-33.6	0.035
AUC_F	17STPAULWW	1473	3	C3:P12817456	12817456	12692687	12944381	-35.8	0.058
AUC_F	17STPAULWW	1473	3	C3:P153124599	153124599	152855739	153393459	39.5	0.049
AUC_F	17STPAULWW	1473	3	C3:P161415134	161415134	157704954	163698154	38.5	0.049
AUC_F	17STPAULWW	1473	3	C3:P178196984	178196984	171315902	178558388	-30.7	0.061
AUC_F	17STPAULWW	1473	4	C4:P22771882	22771882	22483897	23059867	45.5	0.061
AUC_F	17STPAULWW	1473	5	C5:P19149346	19149346	19116963	19181729	44.9	0.035
AUC_F	17STPAULWW	1473	5	C5:P201024607	201024607	200461747	201368901	-34.7	0.033
AUC_F	17STPAULWW	1473	8	C8:P145987724	145987724	144393530	149758442	-45.0	0.041
AUC_F	17STPAULWW	1473	9	C9:P154486630	154486630	154457277	154503425	-30.3	0.036
AUC_F	17STPAULWD	1526	1	C1:P35943782	35943782	35850128	36037436	-51.9	0.030
AUC_F	17STPAULWD	1526	1	C1:P46818021	46818021	46686790	46950585	36.8	0.019
AUC_F	17STPAULWD	1526	1	C1:P288692039	288692039	288572768	289743062	-48.8	0.045

AUC$_F$	17STPAULWD	1526	1	C1:P301650736	301650736	301605345	301713806	-55.2	0.069
AUC$_F$	17STPAULWD	1526	2	C2:P40568132	40568132	40348357	40787907	45.4	0.039
AUC$_F$	17STPAULWD	1526	3	C3:P12817456	12817456	12692687	12944381	-48.6	0.070
AUC$_F$	17STPAULWD	1526	3	C3:P161415134	161415134	157704954	163698154	57.6	0.050
AUC$_F$	17STPAULWD	1526	3	C3:P163834567	163834567	163435493	164233641	-39.6	0.009
AUC$_F$	17STPAULWD	1526	3	C3:P178196984	178196984	171315902	178558388	-33.8	0.049
AUC$_F$	17STPAULWD	1526	4	C4:P22771882	22771882	22483897	23059867	39.9	0.041
AUC$_F$	17STPAULWD	1526	5	C5:P19149346	19149346	19116963	19181729	53.5	0.034
AUC$_F$	17STPAULWD	1526	5	C5:P201024607	201024607	200461747	201368901	-40.7	0.035
AUC$_F$	17STPAULWD	1526	8	C8:P119336307	119336307	112487560	126411262	31.9	0.041
AUC$_F$	17STPAULWD	1526	8	C8:P145987724	145987724	144393530	149758442	-45.0	0.038

(F) AUC$_S$: Area under the curve during the senescence phase

Trait	Environment	Mean	Chr	SNP	Position	Start	End	Effect	r^2
AUC$_S$	16STPAULWW	1608	1	C1:P45444623	45444623	45338853	46123588	29.1	0.019
AUC$_S$	16STPAULWW	1608	1	C1:P279923637	279923637	279588460	280258911	-26.8	0.067
AUC$_S$	16STPAULWW	1608	3	C3:P598760	598760	405961	615334	-21.2	0.044
AUC$_S$	16STPAULWW	1608	3	C3:P19131545	19131545	18590552	20783187	38.7	0.059
AUC$_S$	16STPAULWW	1608	3	C3:P156427376	156427376	155990131	156762895	19.3	0.061
AUC$_S$	16STPAULWW	1608	3	C3:P171558866	171558866	171436197	171681535	22.2	0.037
AUC$_S$	16STPAULWW	1608	4	C4:P183322522	183322522	182200193	183857724	-34.7	0.058
AUC$_S$	16STPAULWW	1608	4	C4:P189987121	189987121	189793789	190180453	-33.8	0.068
AUC$_S$	16STPAULWW	1608	5	C5:P41051272	41051272	40400420	41702652	18.8	0.037
AUC$_S$	16STPAULWW	1608	5	C5:P86743274	86743274	86586629	87103759	26.9	0.048
AUC$_S$	16STPAULWW	1608	5	C5:P195771593	195771593	195676436	195897593	16.4	0.014
AUC$_S$	16STPAULWW	1608	8	C8:P120807215	120807215	120685279	121259449	34.6	0.064
AUC$_S$	16STPAULWW	1608	9	C9:P156684586	156684586	156674600	156694572	21.1	0.059
AUC$_S$	16STPAULWD	1469	1	C1:P279923637	279923637	279588460	280258911	-27.8	0.055
AUC$_S$	16STPAULWD	1469	2	C2:P23201939	23201939	23153799	23250729	26.8	0.063
AUC$_S$	16STPAULWD	1469	3	C3:P19131545	19131545	18590552	20783187	49.0	0.068
AUC$_S$	16STPAULWD	1469	3	C3:P171558866	171558866	171436197	171681535	25.7	0.053
AUC$_S$	16STPAULWD	1469	3	C3:P178242266	178242266	163363932	178970772	18.5	0.034
AUC$_S$	16STPAULWD	1469	5	C5:P86743274	86743274	86586629	87103759	19.2	0.025
AUC$_S$	16STPAULWD	1469	5	C5:P195771593	195771593	195676436	195897593	16.2	0.015
AUC$_S$	16STPAULWD	1469	6	C6:P160999819	160999819	160964454	161035451	44.6	0.058
AUC$_S$	16STPAULWD	1469	6	C6:P168461755	168461755	168445181	168478986	18.9	0.022
AUC$_S$	16STPAULWD	1469	8	C8:P120807215	120807215	120685279	121259449	42.8	0.067
AUC$_S$	16STPAULWD	1469	9	C9:P156684586	156684586	156674600	156694572	20.7	0.052
AUC$_S$	17STPAULWW	1554	1	C1:P17974881	17974881	17904876	18044886	-30.6	0.031
AUC$_S$	17STPAULWW	1554	1	C1:P45444623	45444623	45338853	46123588	57.6	0.058
AUC$_S$	17STPAULWW	1554	1	C1:P279923637	279923637	279588460	280258911	-27.4	0.043
AUC$_S$	17STPAULWW	1554	2	C2:P41103887	41103887	40702422	41307170	36.0	0.065
AUC$_S$	17STPAULWW	1554	3	C3:P11580838	11580838	11521986	11641644	-21.9	0.049
AUC$_S$	17STPAULWW	1554	3	C3:P19131545	19131545	18590552	20783187	46.9	0.053
AUC$_S$	17STPAULWW	1554	3	C3:P156427376	156427376	155990131	156762895	37.8	0.045
AUC$_S$	17STPAULWW	1554	3	C3:P171558866	171558866	171436197	171681535	28.9	0.029
AUC$_S$	17STPAULWW	1554	3	C3:P178242266	178242266	163363932	178970772	35.3	0.058
AUC$_S$	17STPAULWW	1554	5	C5:P27897274	27897274	27791159	28003389	24.4	0.009
AUC$_S$	17STPAULWW	1554	5	C5:P86743274	86743274	86586629	87103759	23.9	0.007
AUC$_S$	17STPAULWW	1554	5	C5:P195771593	195771593	195676436	195897593	22.5	0.018
AUC$_S$	17STPAULWW	1554	7	C7:P22679586	22679586	22230889	23033446	38.5	0.058
AUC$_S$	17STPAULWW	1554	8	C8:P135876370	135876370	134547973	138130347	52.7	0.078
AUC$_S$	17STPAULWW	1554	9	C9:P21726094	21726094	21618509	21833679	-31.8	0.016
AUC$_S$	17STPAULWD	1560	1	C1:P17974881	17974881	17904876	18044886	-38.6	0.032
AUC$_S$	17STPAULWD	1560	1	C1:P38380543	38380543	38295132	38465954	40.0	0.042
AUC$_S$	17STPAULWD	1560	1	C1:P45444623	45444623	45338853	46123588	61.8	0.043

AUC_S	17STPAULWD	1560	1	C1:P57391598	57391598	57087893	61101718	39.5	0.027
AUC_S	17STPAULWD	1560	2	C2:P41103887	41103887	40702422	41307170	40.6	0.040
AUC_S	17STPAULWD	1560	3	C3:P598760	598760	405961	615334	-28.5	0.037
AUC_S	17STPAULWD	1560	3	C3:P11580838	11580838	11521986	11641644	-33.1	0.049
AUC_S	17STPAULWD	1560	3	C3:P156427376	156427376	155990131	156762895	36.7	0.028
AUC_S	17STPAULWD	1560	3	C3:P171558866	171558866	171436197	171681535	34.9	0.021
AUC_S	17STPAULWD	1560	3	C3:P178242266	178242266	163363932	178970772	36.5	0.047
AUC_S	17STPAULWD	1560	5	C5:P27897274	27897274	27791159	28003389	35.9	0.016
AUC_S	17STPAULWD	1560	5	C5:P41051272	41051272	40400420	41702652	39.2	0.066
AUC_S	17STPAULWD	1560	5	C5:P86743274	86743274	86586629	87103759	32.1	0.008
AUC_S	17STPAULWD	1560	5	C5:P195771593	195771593	195676436	195897593	47.0	0.039
AUC_S	17STPAULWD	1560	8	C8:P135876370	135876370	134547973	138130347	83.6	0.066
AUC_S	17STPAULWD	1560	8	C8:P147633339	147633339	147388668	147969727	-37.1	0.035
AUC_S	17STPAULWD	1560	8	C8:P149449909	149449909	149174507	149725311	48.7	0.021
AUC_S	17STPAULWD	1560	8	C8:P149450033	149450033	149174631	149725435	-64.3	0.007
AUC_S	17STPAULWD	1560	9	C9:P21726094	21726094	21618509	21833679	-45.1	0.023

(**G**) GY: Grain yield

Trait	Environment	Mean	Chr	SNP	Position	Start	End	Effect	r^2
GY	16STPAULWW	98.7	1	C1:P105234793	105234793	104033781	106435805	2.00	0.016
GY	16STPAULWW	98.7	1	C1:P287176827	287176827	286675801	288050988	-2.12	0.017
GY	16STPAULWW	98.7	3	C3:P23378661	23378661	18590552	24581733	2.78	0.043
GY	16STPAULWW	98.7	3	C3:P161216584	161216584	160868710	161564458	3.11	0.107
GY	16STPAULWW	98.7	3	C3:P171466075	171466075	169171641	171752186	-1.72	0.011
GY	16STPAULWW	98.7	4	C4:P18518806	18518806	18320663	18716949	-2.01	0.043
GY	16STPAULWW	98.7	4	C4:P223305506	223305506	222020326	224590686	2.26	0.055
GY	16STPAULWW	98.7	5	C5:P10873626	10873626	10862820	11035515	-1.68	0.013
GY	16STPAULWW	98.7	5	C5:P36403315	36403315	36080037	41193941	-1.87	0.035
GY	16STPAULWW	98.7	5	C5:P190151164	190151164	190104240	190198088	-2.25	0.024
GY	16STPAULWW	98.7	5	C5:P210257223	210257223	210241216	210273230	-1.33	0.007
GY	16STPAULWW	98.7	5	C5:P216549115	216549115	216541238	216601720	-2.54	0.052
GY	16STPAULWW	98.7	7	C7:P176278691	176278691	176216146	176341236	-1.21	0.013
GY	16STPAULWW	98.7	8	C8:P11459916	11459916	11400980	11518546	-1.75	0.014
GY	16STPAULWW	98.7	8	C8:P117781798	117781798	117634703	117928893	2.62	0.045
GY	16STPAULWW	98.7	8	C8:P146534702	146534702	146295465	146777002	1.61	0.030
GY	16STPAULWW	98.7	8	C8:P178780270	178780270	178757081	178853608	-1.17	0.009
GY	16STPAULWW	98.7	10	C10:P131303247	131303247	131172449	131608900	1.67	0.059
GY	16STPAULWW	98.7	10	C10:P134585528	134585528	134483455	134687601	-1.73	0.028
GY	16STPAULWW	98.7	10	C10:P149396062	149396062	149353532	149438592	2.63	0.052
GY	16STPAULWD	61.5	1	C1:P72484444	72484444	72191495	72777393	0.99	0.000
GY	16STPAULWD	61.5	1	C1:P73403440	73403440	73118171	73689079	-1.07	0.015
GY	16STPAULWD	61.5	1	C1:P287176827	287176827	286675801	288050988	-1.85	0.027
GY	16STPAULWD	61.5	4	C4:P194554199	194554199	194202320	194906752	1.54	0.040
GY	16STPAULWD	61.5	4	C4:P235217823	235217823	234926588	235509058	1.37	0.073
GY	16STPAULWD	61.5	4	C4:P236473832	236473832	236087967	236702045	1.82	0.006
GY	16STPAULWD	61.5	5	C5:P10873626	10873626	10862820	11035515	-1.23	0.026
GY	16STPAULWD	61.5	5	C5:P18195973	18195973	18169827	18222249	-1.32	0.049
GY	16STPAULWD	61.5	5	C5:P190151164	190151164	190104240	190198088	-1.15	0.017
GY	16STPAULWD	61.5	5	C5:P210257223	210257223	210241216	210273230	-0.90	0.013
GY	16STPAULWD	61.5	5	C5:P216549115	216549115	216541238	216601720	-1.31	0.024
GY	16STPAULWD	61.5	7	C7:P180747435	180747435	180662330	180832615	1.44	0.054
GY	16STPAULWD	61.5	8	C8:P11459916	11459916	11400980	11518546	-1.41	0.019
GY	16STPAULWD	61.5	10	C10:P132244397	132244397	132119122	132369672	-1.88	0.040
GY	17STPAULWW	95.0	1	C1:P105234793	105234793	104033781	106435805	1.38	0.008
GY	17STPAULWW	95.0	1	C1:P288486958	288486958	288365361	288608555	-1.57	0.032
GY	17STPAULWW	95.0	3	C3:P171466075	171466075	169171641	171752186	-2.38	0.038

GY	17STPAULWW	95.0	3	C3:P173523614	173523614	173429947	175933373	-1.40	0.040
GY	17STPAULWW	95.0	4	C4:P166816571	166816571	166543088	167090054	1.53	0.055
GY	17STPAULWW	95.0	4	C4:P194554199	194554199	194202320	194906752	1.96	0.042
GY	17STPAULWW	95.0	4	C4:P223305506	223305506	222020326	224590686	1.47	0.043
GY	17STPAULWW	95.0	5	C5:P36403315	36403315	36080037	41193941	-1.77	0.041
GY	17STPAULWW	95.0	5	C5:P196100018	196100018	196003162	196388113	1.67	0.060
GY	17STPAULWW	95.0	5	C5:P209995538	209995538	209977695	210013381	-1.53	0.024
GY	17STPAULWW	95.0	5	C5:P210257223	210257223	210241216	210273230	-1.35	0.008
GY	17STPAULWW	95.0	5	C5:P216549115	216549115	216541238	216601720	-1.74	0.042
GY	17STPAULWW	95.0	6	C6:P151949044	151949044	151893110	152007719	1.30	0.035
GY	17STPAULWW	95.0	7	C7:P176278691	176278691	176216146	176341236	-1.68	0.037
GY	17STPAULWW	95.0	8	C8:P11459916	11459916	11400980	11518546	-1.68	0.024
GY	17STPAULWW	95.0	8	C8:P146534702	146534702	146295465	146777002	1.22	0.019
GY	17STPAULWW	95.0	8	C8:P154557081	154557081	154342412	154771750	1.68	0.030
GY	17STPAULWW	95.0	8	C8:P178780270	178780270	178757081	178853608	-1.54	0.040
GY	17STPAULWD	77.1	1	C1:P73403440	73403440	73118171	73689079	-1.99	0.042
GY	17STPAULWD	77.1	1	C1:P105234793	105234793	104033781	106435805	1.12	0.009
GY	17STPAULWD	77.1	1	C1:P288486958	288486958	288365361	288608555	-2.07	0.025
GY	17STPAULWD	77.1	1	C1:P301650736	301650736	301605345	301713806	-2.01	0.021
GY	17STPAULWD	77.1	3	C3:P161216584	161216584	160868710	161564458	1.34	0.012
GY	17STPAULWD	77.1	3	C3:P171466075	171466075	169171641	171752186	-1.53	0.008
GY	17STPAULWD	77.1	3	C3:P221445068	221445068	221014376	222462406	-2.07	0.054
GY	17STPAULWD	77.1	4	C4:P194554199	194554199	194202320	194906752	2.83	0.029
GY	17STPAULWD	77.1	5	C5:P18195973	18195973	18169827	18222249	-2.08	0.078
GY	17STPAULWD	77.1	5	C5:P190151164	190151164	190104240	190198088	-1.77	0.042
GY	17STPAULWD	77.1	5	C5:P196100018	196100018	196003162	196388113	1.94	0.052
GY	17STPAULWD	77.1	5	C5:P210257223	210257223	210241216	210273230	-2.42	0.032
GY	17STPAULWD	77.1	6	C6:P95684075	95684075	95458100	95946296	1.56	0.032
GY	17STPAULWD	77.1	6	C6:P151949044	151949044	151893110	152007719	1.53	0.029
GY	17STPAULWD	77.1	7	C7:P165934560	165934560	165873173	165995947	1.80	0.019
GY	17STPAULWD	77.1	7	C7:P176278691	176278691	176216146	176341236	-1.31	0.017
GY	17STPAULWD	77.1	8	C8:P154557081	154557081	154342412	154771750	2.89	0.051
GY	17STPAULWD	77.1	9	C9:P150712517	150712517	150685341	150745562	-1.56	0.038
GY	17STPAULWD	77.1	10	C10:P131303247	131303247	131172449	131608900	1.19	0.023

(**H**) KN: Kernel number

Trait	Environment	Mean	Chr	SNP	Position	Start	End	Effect	r^2
KN	16STPAULWW	3681	1	C1:P269190758	269190758	268809671	269571845	-96.2	0.055
KN	16STPAULWW	3681	2	C2:P25984668	25984668	25919325	26050011	64.0	0.053
KN	16STPAULWW	3681	3	C3:P23794554	23794554	23162883	24581733	103.7	0.059
KN	16STPAULWW	3681	3	C3:P33251985	33251985	31710620	34403914	91.3	0.059
KN	16STPAULWW	3681	4	C4:P13038563	13038563	12913246	13163880	92.8	0.052
KN	16STPAULWW	3681	5	C5:P18195973	18195973	18169827	18222249	-70.6	0.032
KN	16STPAULWW	3681	6	C6:P108912978	108912978	108829085	109047002	-109.2	0.057
KN	16STPAULWW	3681	6	C6:P124820061	124820061	124658473	126217177	-100.5	0.056
KN	16STPAULWW	3681	7	C7:P834918	834918	806627	849528	-71.7	0.051
KN	16STPAULWW	3681	8	C8:P126889655	126889655	126773312	127002801	99.0	0.047
KN	16STPAULWD	2904	1	C1:P269190758	269190758	268809671	269571845	-57.3	0.031
KN	16STPAULWD	2904	5	C5:P18195973	18195973	18169827	18222249	-75.9	0.057
KN	16STPAULWD	2904	6	C6:P124820061	124820061	124658473	126217177	-58.5	0.032
KN	17STPAULWW	3401	3	C3:P23794554	23794554	23162883	24581733	73.8	0.036
KN	17STPAULWW	3401	5	C5:P18195973	18195973	18169827	18222249	-77.9	0.054
KN	17STPAULWW	3401	6	C6:P108912978	108912978	108829085	109047002	-77.1	0.032
KN	17STPAULWW	3401	6	C6:P124820061	124820061	124658473	126217177	-69.5	0.033
KN	17STPAULWD	2679	3	C3:P23794554	23794554	23162883	24581733	58.3	0.020

KN	17STPAULWD	2679	5	C5:P18195973	18195973	18169827	18222249	-87.8	0.079
KN	17STPAULWD	2679	5	C5:P50414066	50414066	50139931	50700049	82.6	0.078

(**I**) TKW: Thousand kernel weight

Trait	Environment	Mean	Chr	SNP	Position	Start	End	Effect	r^2
TKW	16STPAULWW	272	4	C4:P174203978	174203978	173932121	174845693	5.75	0.083
TKW	16STPAULWW	272	4	C4:P201810967	201810967	200479742	203404742	-5.57	0.073
TKW	16STPAULWW	272	8	C8:P138313489	138313489	138045988	139924458	-11.64	0.067
TKW	16STPAULWD	209	4	C4:P174203978	174203978	173932121	174845693	3.38	0.036
TKW	17STPAULWW	289	4	C4:P174203978	174203978	173932121	174845693	4.13	0.051
TKW	17STPAULWW	289	4	C4:P201810967	201810967	200479742	203404742	-4.94	0.053
TKW	17STPAULWD	285	4	C4:P174203978	174203978	173932121	174845693	4.16	0.052
TKW	17STPAULWD	285	4	C4:P201810967	201810967	200479742	203404742	-4.10	0.039

(**J**) FF: Female flowering

Trait	Environment	Mean	Chr	SNP	Position	Start	End	Effect	r^2
FF	16STPAULWW	1002	3	C3:P156803877	156803877	156455198	157152933	-8.31	0.070
FF	16STPAULWW	1002	3	C3:P163388470	163388470	163080341	163698154	7.36	0.066
FF	16STPAULWW	1002	4	C4:P144897625	144897625	143733873	146009071	6.75	0.059
FF	16STPAULWW	1002	5	C5:P3897375	3897375	3888640	3909325	4.58	0.012
FF	16STPAULWW	1002	7	C7:P22679586	22679586	22230889	23033446	6.33	0.020
FF	16STPAULWW	1002	8	C8:P126884646	126884646	121631062	130317984	8.19	0.070
FF	16STPAULWW	1002	8	C8:P146534702	146534702	146292401	149758442	4.83	0.065
FF	16STPAULWW	1002	9	C9:P103416791	103416791	103001713	106828573	6.57	0.035
FF	16STPAULWD	1023	1	C1:P289632477	289632477	288376525	289743062	-6.78	0.038
FF	16STPAULWD	1023	3	C3:P156803877	156803877	156455198	157152933	-5.50	0.041
FF	16STPAULWD	1023	3	C3:P163388470	163388470	163080341	163698154	5.85	0.058
FF	16STPAULWD	1023	4	C4:P144897625	144897625	143733873	146009071	7.42	0.066
FF	16STPAULWD	1023	4	C4:P245200284	245200284	244768961	245243661	-6.05	0.021
FF	16STPAULWD	1023	5	C5:P219970981	219970981	219962936	219979031	-4.17	0.018
FF	16STPAULWD	1023	7	C7:P22679586	22679586	22230889	23033446	7.02	0.023
FF	16STPAULWD	1023	8	C8:P93156619	93156619	92970781	98630561	7.26	0.067
FF	16STPAULWD	1023	8	C8:P126884646	126884646	121631062	130317984	5.30	0.047
FF	17STPAULWW	921	1	C1:P276928107	276928107	276483051	277373163	-6.86	0.036
FF	17STPAULWW	921	1	C1:P279600761	279600761	279249663	279951859	3.91	0.000
FF	17STPAULWW	921	2	C2:P38088289	38088289	37737045	38442684	3.80	0.052
FF	17STPAULWW	921	3	C3:P17436974	17436974	16362424	22890362	5.12	0.037
FF	17STPAULWW	921	3	C3:P156803877	156803877	156455198	157152933	-3.51	0.043
FF	17STPAULWW	921	3	C3:P163388470	163388470	163080341	163698154	4.62	0.060
FF	17STPAULWW	921	4	C4:P144897625	144897625	143733873	146009071	2.72	0.026
FF	17STPAULWW	921	4	C4:P245200284	245200284	244768961	245243661	-5.32	0.039
FF	17STPAULWW	921	5	C5:P3897375	3897375	3888640	3909325	2.74	0.012
FF	17STPAULWW	921	5	C5:P219970981	219970981	219962936	219979031	-2.58	0.010
FF	17STPAULWW	921	7	C7:P22679586	22679586	22230889	23033446	5.04	0.026
FF	17STPAULWW	921	7	C7:P133687871	133687871	133526699	133896563	4.59	0.024
FF	17STPAULWW	921	8	C8:P126884646	126884646	121631062	130317984	5.06	0.069
FF	17STPAULWW	921	8	C8:P146534702	146534702	146292401	149758442	4.85	0.103
FF	17STPAULWW	921	8	C8:P155674123	155674123	155478177	155854299	-4.23	0.061
FF	17STPAULWW	921	9	C9:P103416791	103416791	103001713	106828573	4.61	0.061
FF	17STPAULWW	921	9	C9:P118646113	118646113	118528918	118763308	3.37	0.032
FF	17STPAULWW	921	10	C10:P1047681	1047681	927762	1077574	4.27	0.037
FF	17STPAULWW	921	10	C10:P92756648	92756648	91684251	101453608	-5.93	0.018
FF	17STPAULWW	921	10	C10:P149288065	149288065	149245908	149330222	-2.52	0.009

FF	17STPAULWD	966	3	C3:P5166485	5166485	5140256	5193137	6.41	0.038
FF	17STPAULWD	966	3	C3:P17436974	17436974	16362424	22890362	9.61	0.043
FF	17STPAULWD	966	3	C3:P156803877	156803877	156455198	157152933	-3.30	0.052
FF	17STPAULWD	966	3	C3:P157314938	157314938	156965838	157901230	-8.19	0.092
FF	17STPAULWD	966	3	C3:P163388470	163388470	163080341	163698154	3.02	0.040
FF	17STPAULWD	966	5	C5:P3897375	3897375	3888640	3909325	4.69	0.027
FF	17STPAULWD	966	5	C5:P219970981	219970981	219962936	219979031	-4.14	0.023
FF	17STPAULWD	966	6	C6:P152874859	152874859	152561367	153100500	-4.63	0.045
FF	17STPAULWD	966	7	C7:P22679586	22679586	22230889	23033446	5.52	0.017
FF	17STPAULWD	966	8	C8:P126884646	126884646	121631062	130317984	6.08	0.059
FF	17STPAULWD	966	9	C9:P9867396	9867396	9836653	9898139	4.24	0.038
FF	17STPAULWD	966	9	C9:P110232248	110232248	84055235	111174995	-7.61	0.059
FF	17STPAULWD	966	9	C9:P116790604	116790604	116705682	116855189	4.75	0.046
FF	17STPAULWD	966	10	C10:P92756648	92756648	91684251	101453608	-13.02	0.049

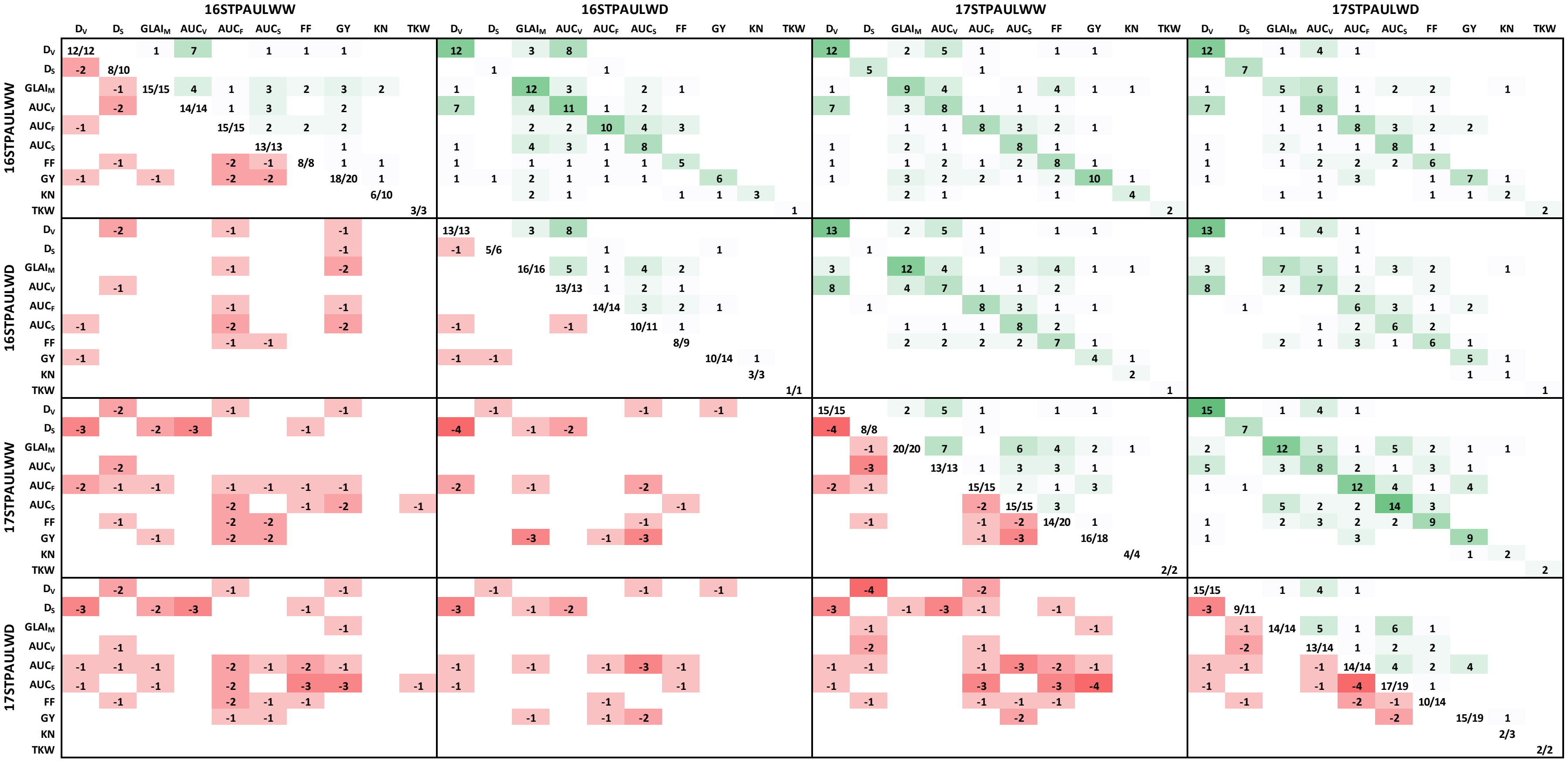

Fig. S6 Colocalization analysis of detected QTLs. The number of colocalizing QTLs for a pair of traits is reported in the corresponding box. Colocalizations between QTLs whose reference alleles have effects of the same sign are reported in the upper triangle in green. QTLs with opposite sign effects are reported in the lower triangle in red. On the diagonal are the ratios between the number of QTLs with at least one colocalization (whether with the same trait in another trial or with a different trait) and the total number of QTLs detected for this trait in this environment.

(**A**) D_V: Duration of the vegetative phase

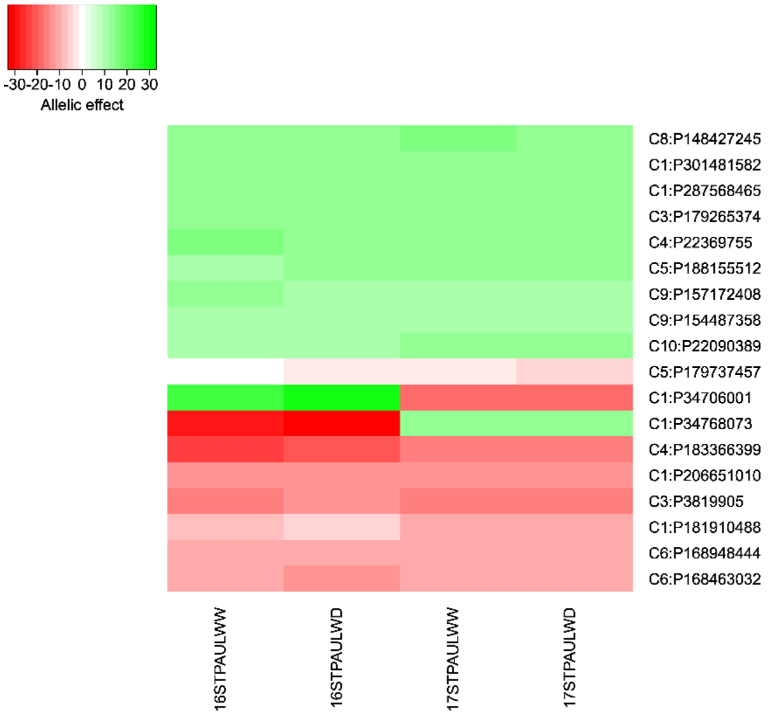

(**B**) D_S: Duration of the senescence phase

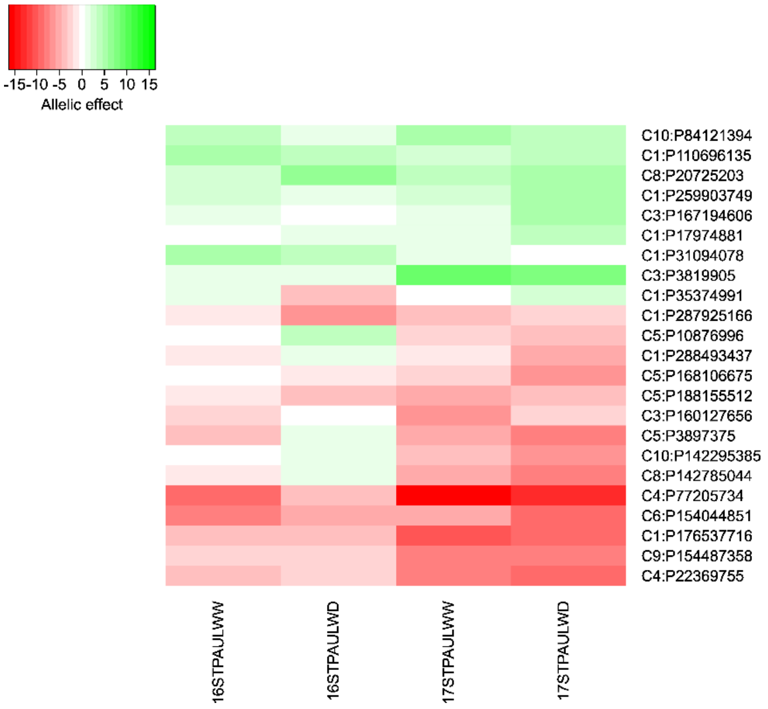

(**C**) GLAI$_M$: Maximum GLAI

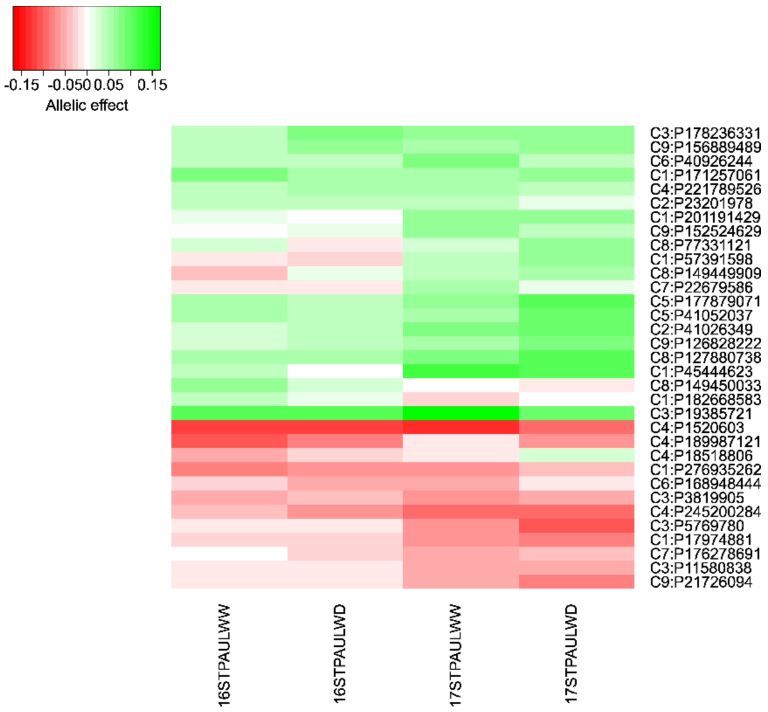

(**D**) AUC$_V$: Area under the curve during the vegetative phase

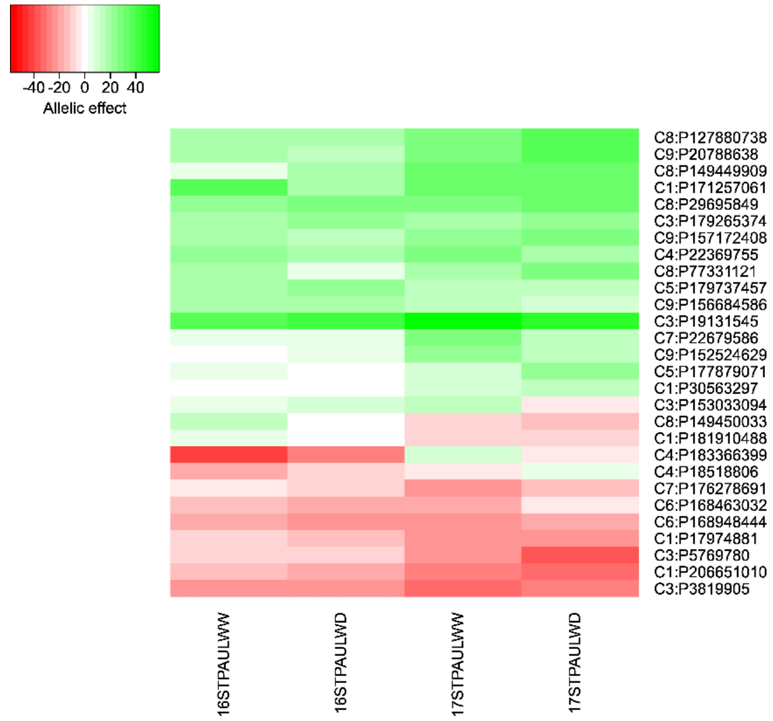

(**E**) AUC$_F$: Area under the curve during the flowering phase

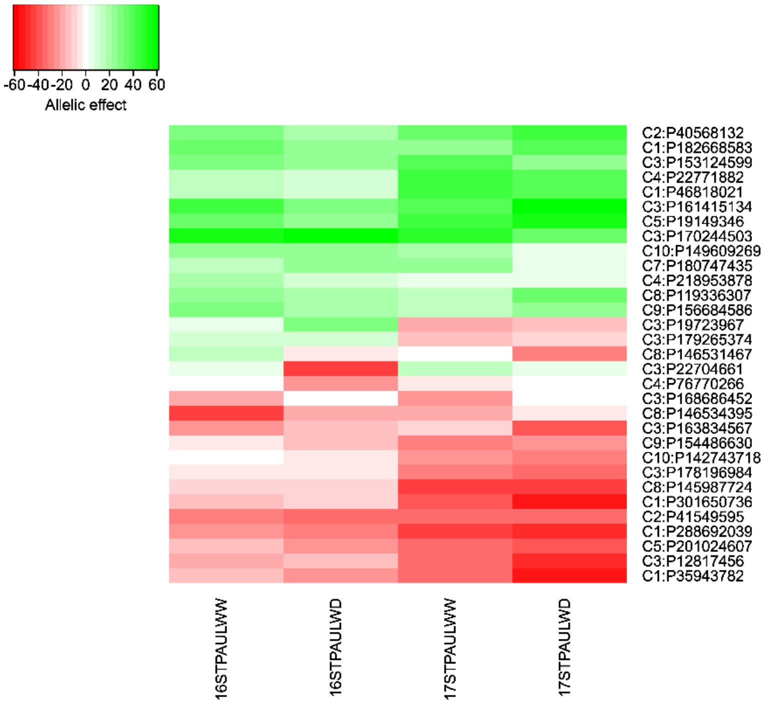

(**F**) AUC$_S$: Area under the curve during the senescence phase

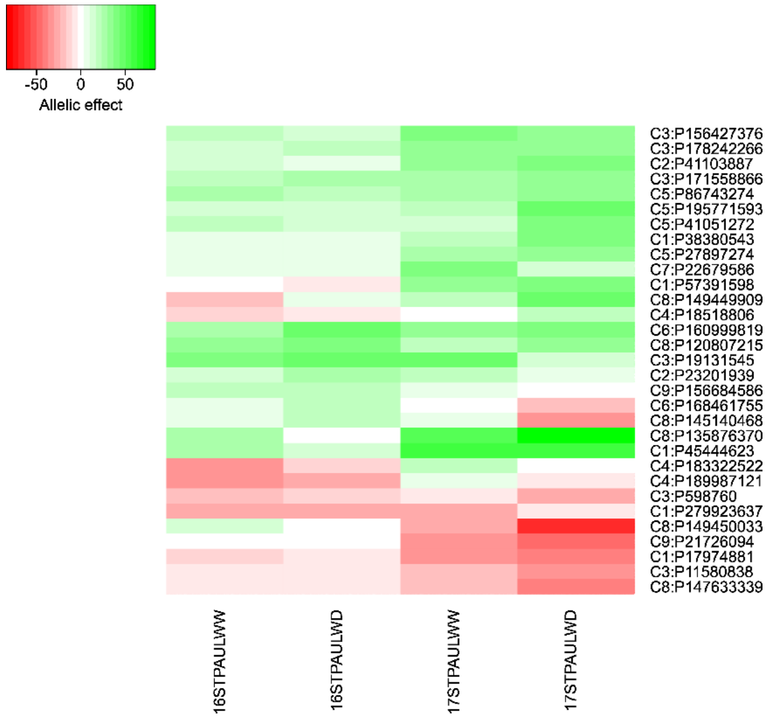

(**G**) GY: Grain yield

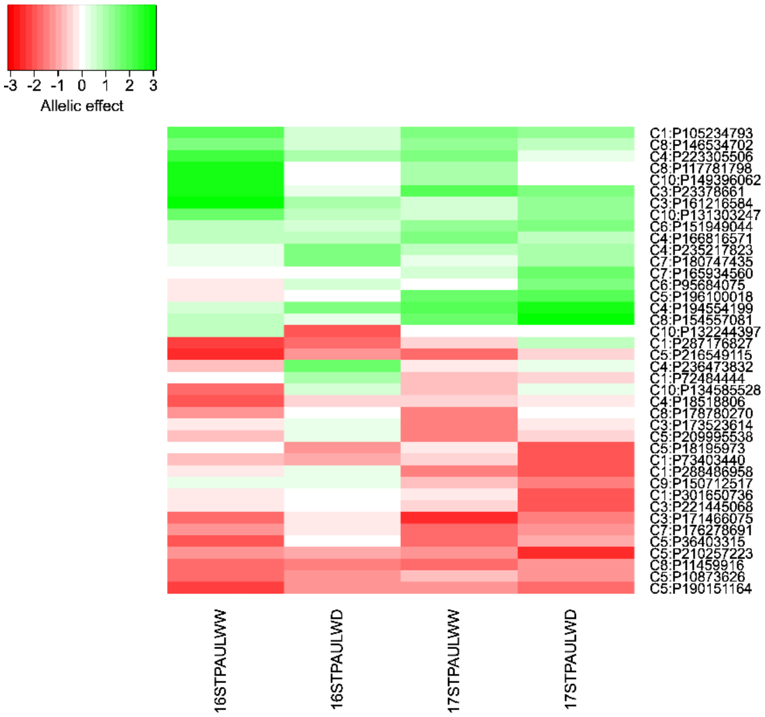

(**H**) KN: Kernel number

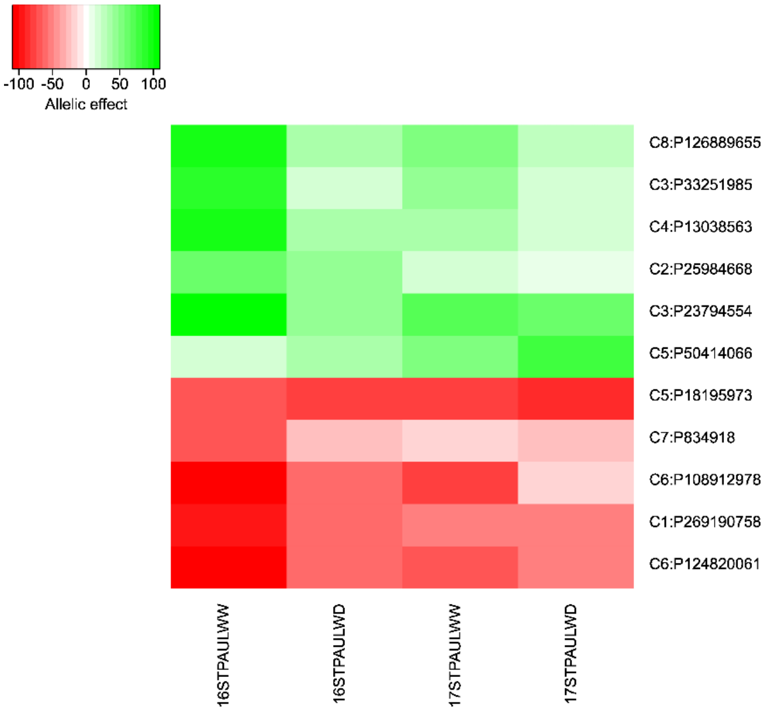

(**I**) TKW: Thousand kernel weight

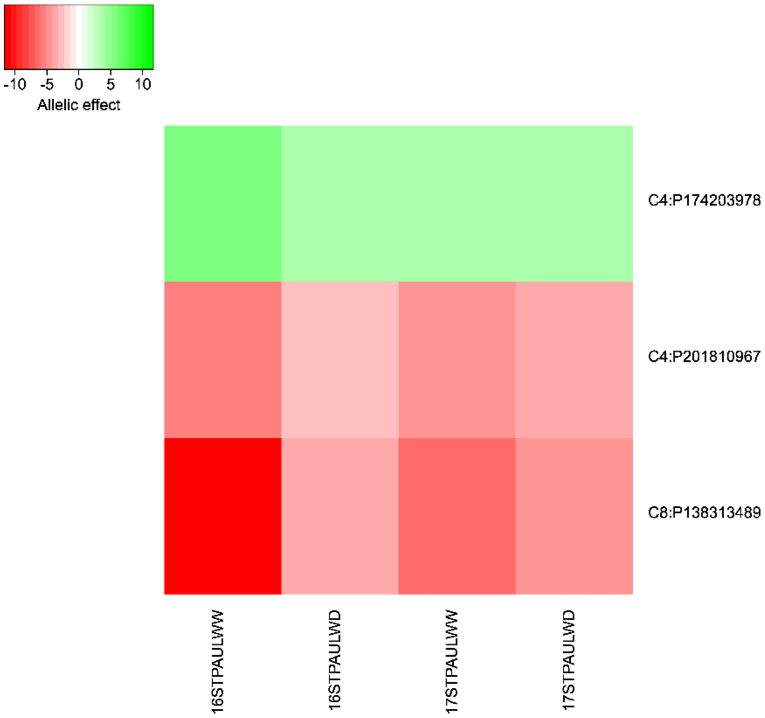

(**J**) FF: Female flowering

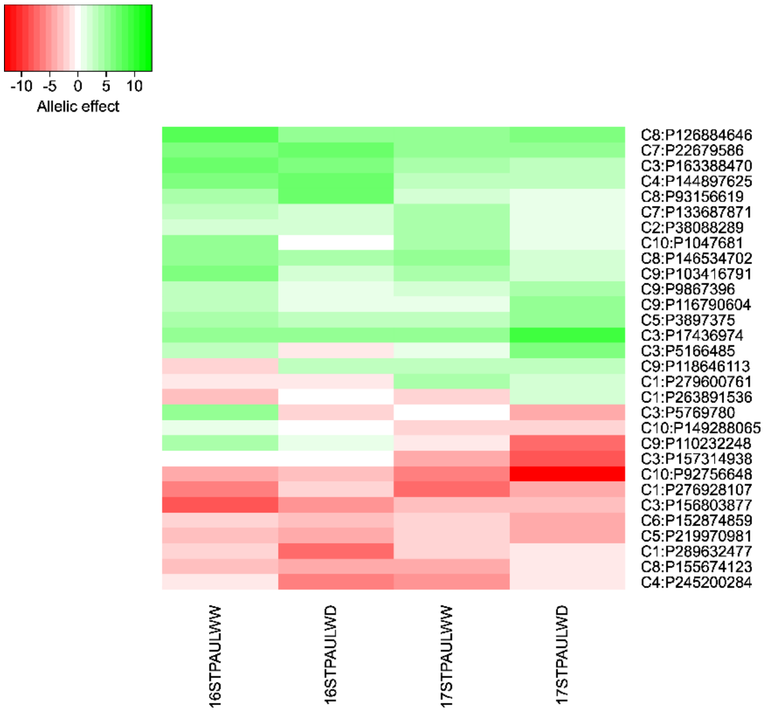

Fig. S7 QTL allelic effects in the four environments in which 324 maize hybrids were evaluated. The effects are those of the reference allele from the B73 genome

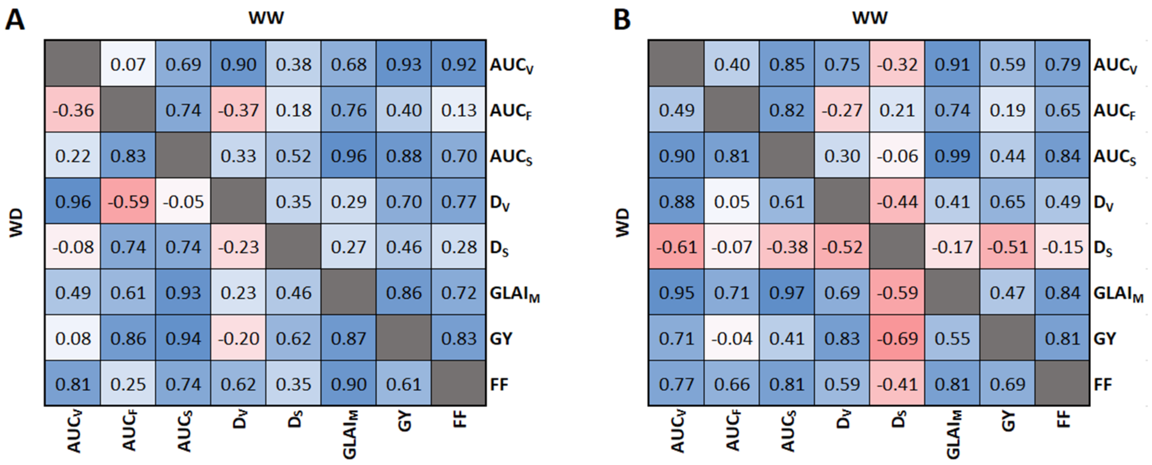

Fig. S8 Genetic correlations between the 6 GLAI traits, GY and FF. (**A**) 16STPAUL, (**B**) 17STPAUL. The upper triangle presents the genetic correlation coefficient between each pair of traits in the well-watered (WW) condition. Similarly, the lower triangle presents the correlations in the water-deficient (WD) condition. The genetic correlations are obtained from the GEMMA software for the eight traits considered in the M_{MV} approach.

.

Table S4 Variance decomposition of the 18 additional GLAI traits on the trial network. σ_g^2, genetic variance; CV_g, genetic coefficient of variation; σ_{ge}^2, variance of the GxE interaction; σ_{ge}^2/σ_g^2, the ratio of the two variances (in %); H², generalized heritability. The model including GxE interaction for S_{EV} did not converge. ***, *pvalue* ≤ 0.001; **, *pvalue* ≤ 0.01; *, *pvalue* ≤ 0.05; n.s., *pvalue* > 0.05.

Trait	Unit	Min-Max	Mean	σ_g^2		CV_g	σ_{ge}^2		σ_{ge}^2/σ_g^2 (%)	H²
AUC_{EV}	$m^2.m^{-2}.GDD6$	25.6 - 149	64.4	174	***	20.47	4.27	***	2.46	0.90
AUC_{LV}	$m^2.m^{-2}.GDD6$	336 - 1384	780	12656	***	14.43	154	***	1.21	0.89
AUC_{SS}	$m^2.m^{-2}.GDD6$	458 - 1770	1074	7881	***	8.27	751	***	9.53	0.86
AUC_{RS}	$m^2.m^{-2}.GDD6$	223 - 862	515	2083	***	8.86	242	***	11.61	0.86
AUC_C	$m^2.m^{-2}.GDD6$	1563 - 5973	3720	103968	***	8.67	4931	***	4.74	0.87
D_{EV}	GDD6	310 - 491	395	1132	***	8.52	$2.98.10^{-5}$	n.s.	0	0.91
D_{LV}	GDD6	342 - 529	434	1206	***	8	$4.30.10^{-9}$	n.s.	0	0.91
D_F	GDD6	198 - 561	412	2224	***	11.45	270	***	12.15	0.89
D_{SS}	GDD6	250 - 423	374	160	***	3.38	83.7	***	52.47	0.84
D_{RS}	GDD6	339 - 469	416	99.0	***	2.39	74.7	***	75.45	0.82
D_C	GDD6	1626 - 2130	2030	389	***	0.97	984	***	253.24	0.68
S_{EV}	$m^2.m^{-2}.GDD6^{-1}$	$1.94.10^{-4}$ – $1.26.10^{-3}$	$5.55.10^{-4}$	$2.53.10^{-8}$	***	28.66	-		-	0.91
S_{LV}	$m^2.m^{-2}.GDD6^{-1}$	$3.35.10^{-3}$ – $1.24.10^{-2}$	9.10^{-3}	$4.76.10^{-7}$	***	9.73	$1.33.10^{-8}$	***	2.79	0.88
S_{SS}	$m^2.m^{-2}.GDD6^{-1}$	$-2.74.10^{-3}$ - $-4.91.10^{-4}$	$-1.06.10^{-3}$	$3.72.10^{-8}$	***	18.26	$1.11.10^{-8}$	***	29.83	0.86
S_{RS}	$m^2.m^{-2}.GDD6^{-1}$	$-1.31.10^{-2}$ - $-3.13.10^{-3}$	$-6.92.10^{-3}$	$5.82.10^{-7}$	***	11.03	$4.62.10^{-8}$	***	7.95	0.87
D_{25}	GDD6	1089 - 1553	1424	1123	***	2.35	834	***	74.26	0.83
D_{50}	GDD6	860 - 1328	1176	1837	***	3.65	727	***	39.59	0.87
D_{75}	GDD6	621 - 1129	958	3240	***	5.94	744	***	22.95	0.88

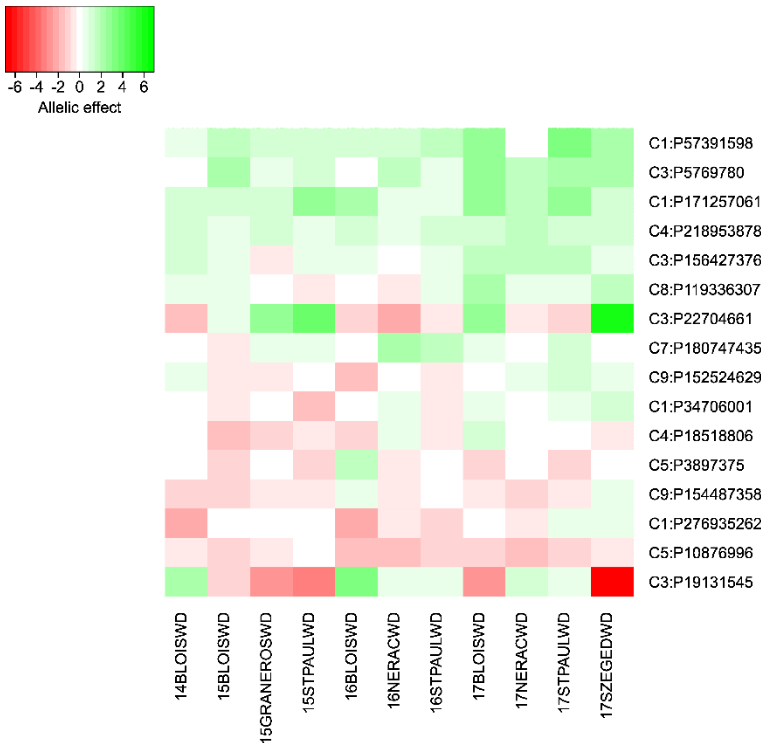

Fig. S9 Allelic effects of GLAI QTLs with significant effect on GY_{11}. The effects are those of the reference allele from the B73 genome.

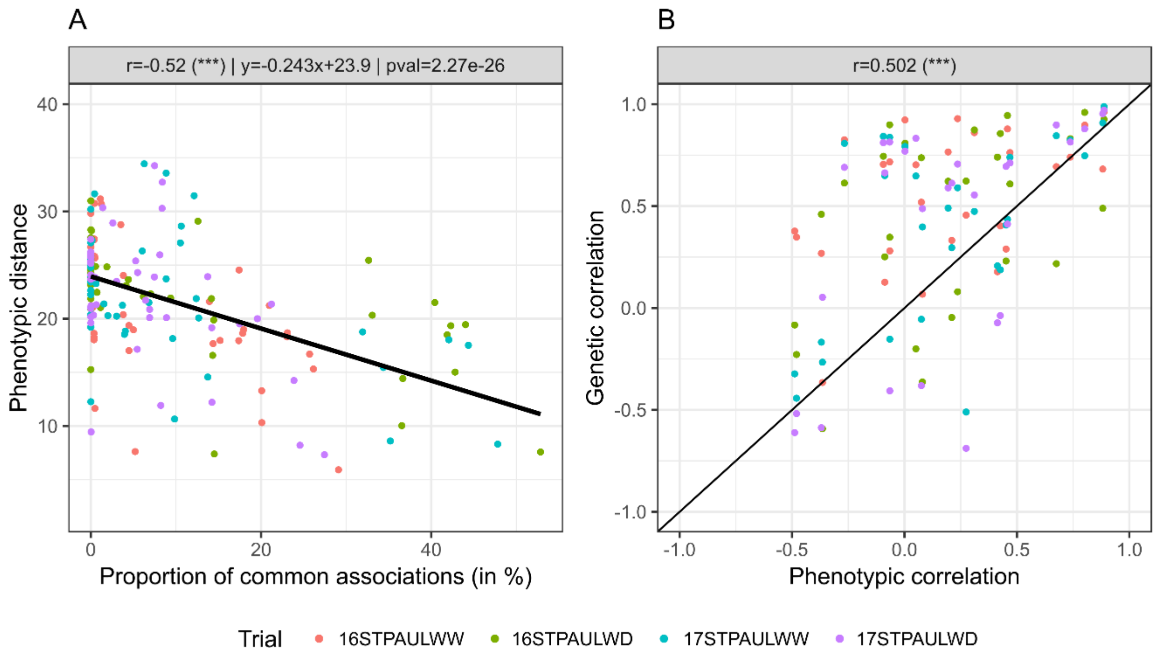

Fig. S10 Consistency between phenotypic link and genetic link. (A) Relationship between the phenotypic distance and the percentage of associations in common for each pair of traits tested in GWAS. The phenotypic distance between each pair of traits was calculated using Ward's distance on the normalized adjusted means. The black slope is the regression line. It appears that the more the phenotypic distance increases, the more the number of common associations (same SNP detected in a given environment) decreases. **(B) Relationship between phenotypic (r_p) and genetic (r_g) correlation.** The phenotypic correlations are the Pearson correlations calculated on the adjusted means, and the genetic correlations are obtained from the GEMMA software for the eight traits considered in the M_{MV} approach. The black slope shows the 1:1 ratio. The significant correlation between r_p and r_g shows that phenotypic correlations can be used as a first approach to estimate genetic correlations.
